# Supplementary material for: Pre-existing retinal inflammation exacerbates gene therapy-associated uveitis in more rapidly progressive retinal degeneration
Source: Mol Ther Adv. 2025 Dec 26;34(1):201652. doi: 10.1016/j.omta.2025.201652 (PMC13370167; doi:10.1016/j.omta.2025.201652)
Supplement: Document S1. Figures S1–S7 and Tables S1–S3 [file mmc1.pdf]

OMTA, Volume *34*

Supplemental information

Pre-existing retinal inflammation exacerbates gene therapy-associated uveitis in more rapidly progressive retinal degeneration

Molly C. John, Cristina Martinez-Fernandez de la Camara, Ahmed Salman, Federica Staurenghi, Hoda Shamsnajafabadi, Michelle E. McClements, Jasmina Cehajic-Kapetanovic, Alissa Bray, M Dominik Fischer, Andrew D. Dick, Robert E. MacLaren, and Kanmin Xue

SUPPLEMENTAL MATERIAL

Table S1 -Primers for AAV Titration qPCR

Primer Name	Sequence	Target
GFPqPCR_F	ACTACCCTGACCTATGGCGT	GFP Transgene
GFPqPCR_R	CTCTCCTGCACATAGCCCT	GFP Transgene

Table S2 - List of markers and fluorophores used flow cytometric analysis of retinal immune cell populations

Target Cell Type	Antibody	Fluorophore	Staining Concentration (ug/ml)
All Leukocytes	CD45 30-F11	Brilliant Violet 711	0.5
Monocytes, Macrophage, dendritic cells, NK cell, granulocytes, B and T cell subsets	CD11b M1/70	APC	0.25
T cells, NK T cells	CD3 17A2	AlexaFluor 700	5
T cell subset	CD4 RM4-5	Brilliant Violet 785	0.5
B cells	CD19 6D5	APC/Fire 750	0.5
Natural Killer Cells	NK-1.1 PK136	PE/Cyanine7	1
Dendritic Cells	CD11c N418	PerCP/Cyanine5.5	1
T Cell subset	CD8a 53-6.7	PE	0.125

***Table S3* - Antibodies used for immunohistochemistry analysis of the retina.**

Antibody	Target	Concentration
Rabbit anti-IBA1 (Wako, WDE1198, 019-19741)	IBA1 - Microglia, Murine	1:500
Rabbit anti-GFAP (ab7260, Abcam)	GFAP - Muller Glia, Murine	1:500
Goat anti-GFAP (ab5353, Abcam)	GFAP - Muller Glia, Murine	1:500
Rat anti-CD45 (30-F11, Biolegend)	CD45 - All immune cells, Murine	1:200
Mouse anti-ZO1 (ZO1-1A12, Invitrogen)	ZO-1 – Tight junctions	1:200
Goat anti-CD68 (sc-7084, Santa Cruz Biotechnology)	CD68 - Microglia, Murine	1:100
Donkey anti-Mouse-AF647 (Biolegend)	Mouse IgG	1:400
Donkey anti-Rabbit-AF568 (Biolegend)	Rabbit IgG	1:400
Goat anti-Rat-AF568 (Biolegend)	Rat IgG	1:400
Donkey ant-Goat-AF647 (Biolegend)	Goat IgG	1:400

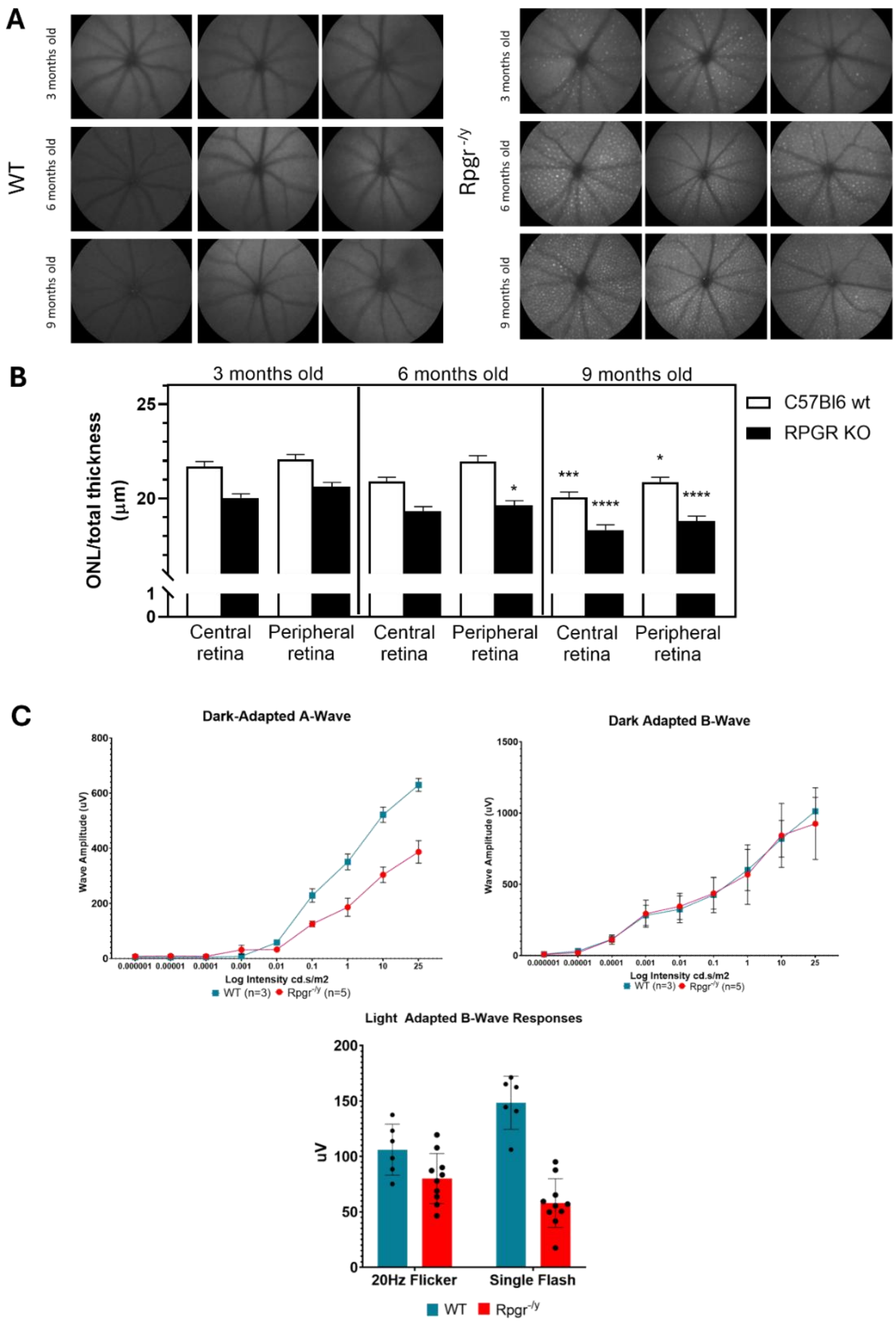

Figure S1. $Rpgr^{-/y}$ progressive phenotype 3-9 months. *(**A**) Progressive development of hyperreflective spots as seen on cSLO in $Rpgr^{-/y}$ versus C57BL6j WT mice from 3 to 9 months of age. (**B**) Progressive thinning of $Rpgr^{-/y}$ retinae from 3-9 months of age. Significance versus the same strain at 3 months of age determined by ANOVA (* p<0.05, *** p<0.001, **** p<0.0001). (**C**) Electroretinography assessment of 12-month old $Rpgr^{-/y}$ mice (n=5) versus age-matched controls (n=3).*

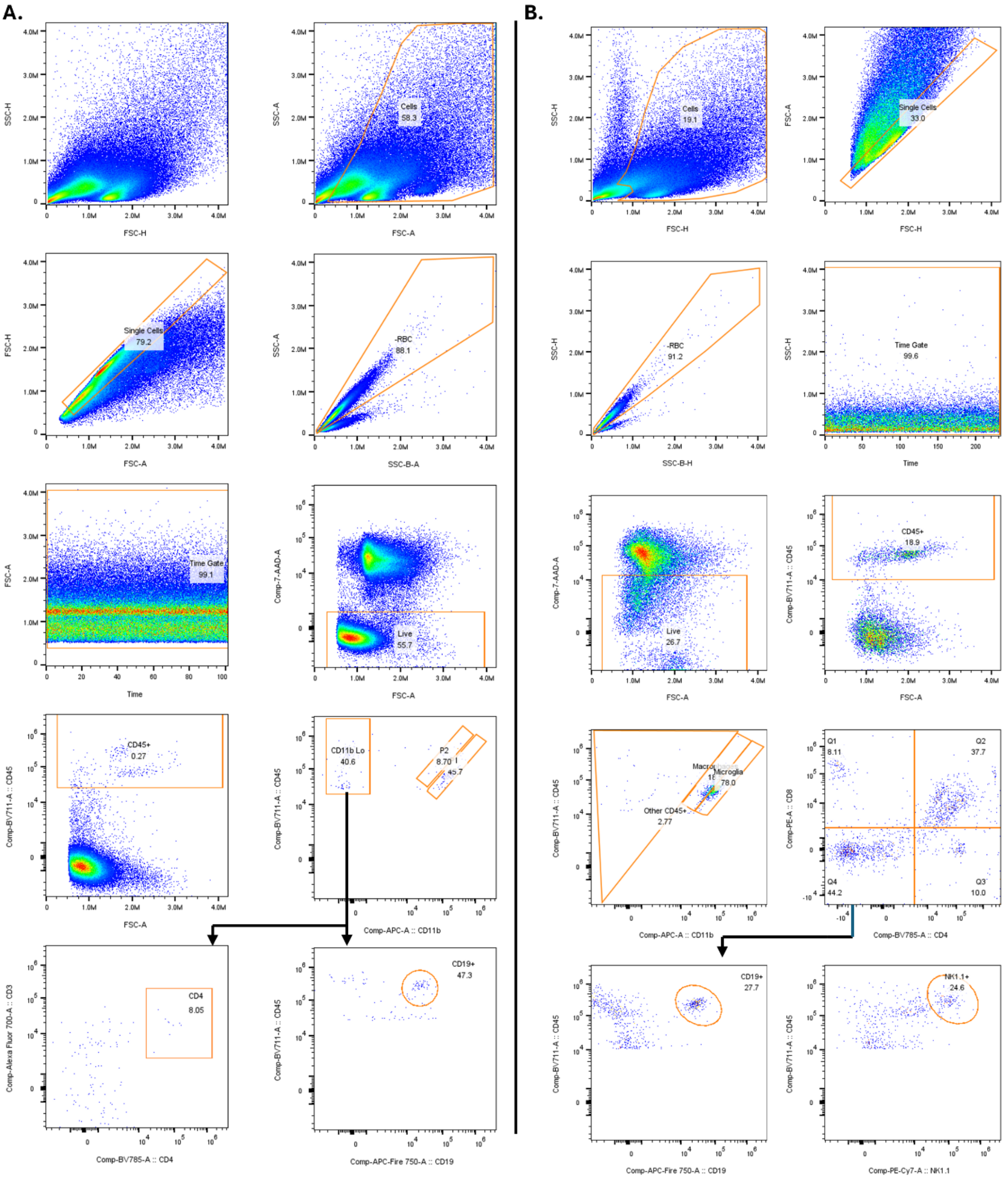

Figure S2. Rpgr$^{-/y}$ and Rho^{P23H} Immunotyping Gating Strategy. *Gating was performed subjectively on multicolour stained retinal samples from Rpgr$^{-/y}$, Rho^{P23H} and WT animals. (**A**) Rpgr$^{-/y}$ and corresponding WT gating. The same gates were applied across all samples, the gated population was brought forward into the next consecutive gate with the exception of the CD4 and CD19 gating which was performed on the population in the 'CD11b Lo' gate as indicated. (**B**) Rho^{P23H} and corresponding WT gating. The same gates were applied across all samples, the gated population was brought forward into the next consecutive gate unless stated. CD19 gating was performed on the population in the 'CD4-CD8-' Q4 gate as indicated. NK1.1 gating taken from a 'NOT' gate of CD19. To allow gating of rare events, gates were decided using a concated sample of all CD45 events in all samples. Gate statistics shown represent the proportion of plotted events within each gate. Gating analysis was performed in FlowJo v10.10.0*

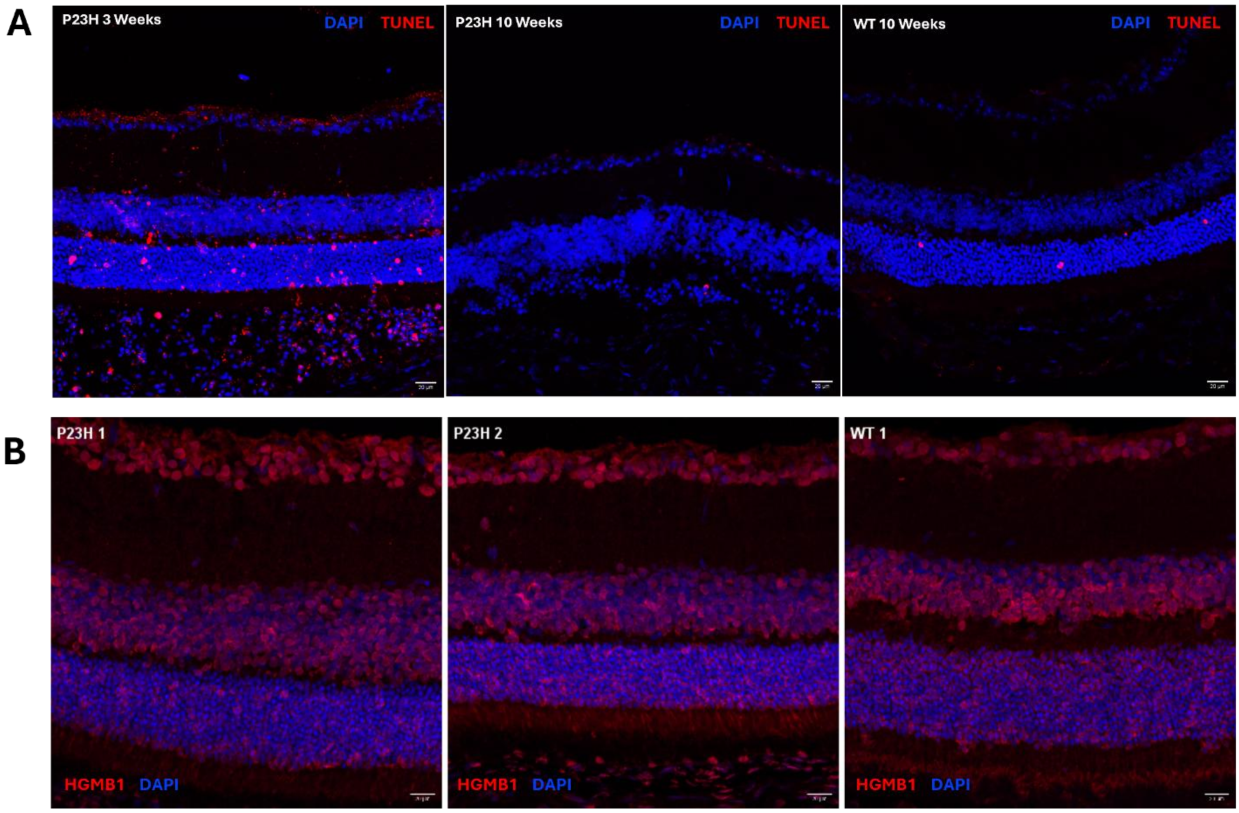

Figure S3. Apoptosis in Rho^{P23H} 3 and 10 week old mice and Rho^{P23H} HGMB1 Staining. (**A**) TUNEL staining in two representative Rho^{P23H} retinas at 3-weeks versus 10-weeks old shows extensive outer retina TUNEL positivity in the younger mice that decreases in older animals, combined with a loss of the ONL. (**B**) HGMB1 staining in two representative Rho^{P23H} retinas (3-weeks) versus the wild-type.

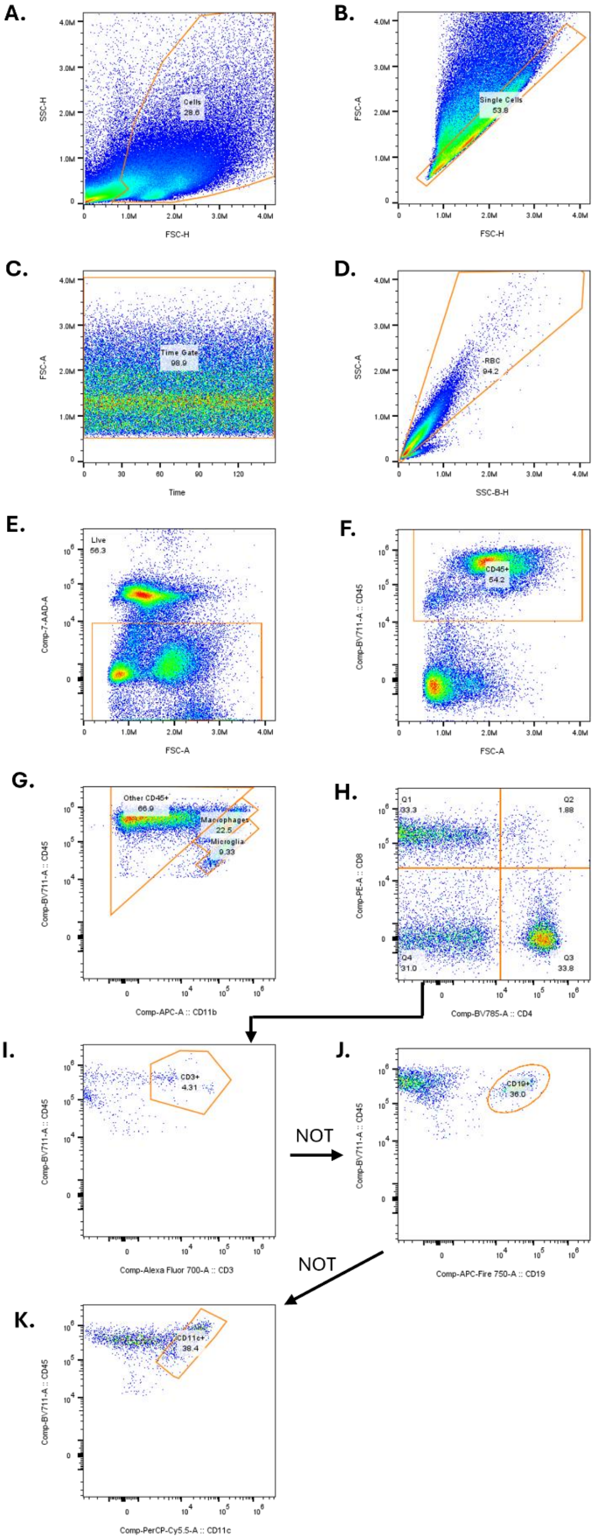

Figure S4. Gating Strategy for AAV treated Rpgr and WT samples. *Gating was performed subjectively on multicolor stained retinal samples from Rpgr and WT animals. The same gates were applied across all samples. In plots A-H the gated population was brought forward into the nest consecutive gate. In plots I gating was performed on the population in the CD4-CD8- 'Q4' gate from plot H as indicated. Plot J was taken from a 'NOT' gate of plot I. Plot K was taken from a NOT gate of plot J. Gate statistics shown represent the proportion of plotted events within each gate. Gating analysis was performed in FlowJo v10.10.0*

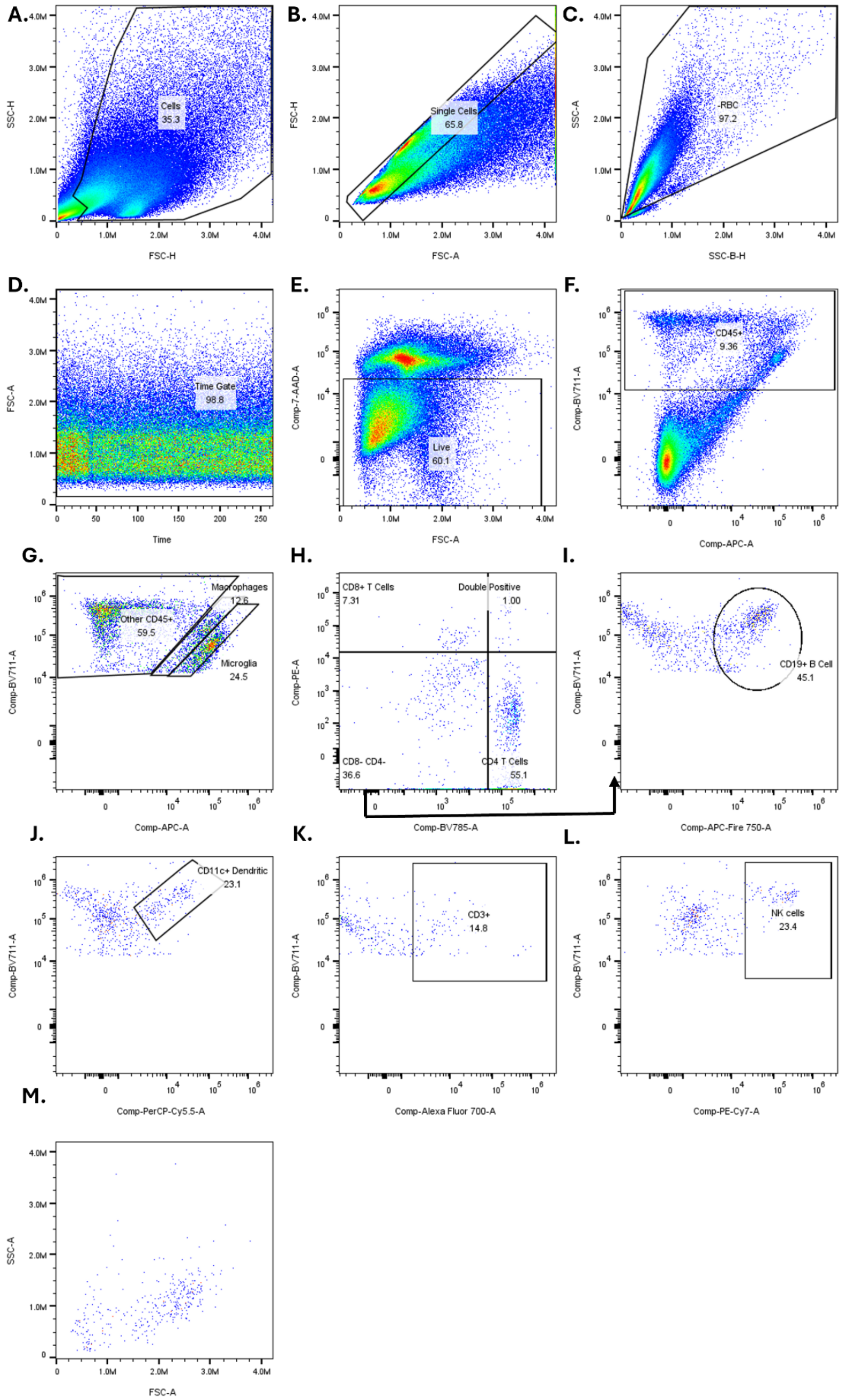

Figure S5. AAV Treated P23H Immunotyping Gating Strategy. *Gating was performed subjectively on multicolor stained retinal samples from AAV or PBS treated retinas from P23H and WT animals. The same gates were applied across all samples. In plots A-H the gated population was brought forward into the next consecutive gate. In plot I gating was performed on the population in the CD4-CD8- 'Q4' gate from plot H as indicated. Plots J-M were taken from a 'NOT' gate of the gated populations on preceding plot. Plot M gives the forward and side scatter profile of untyped CD45 positive cells that did not fit other gates. Gate statistics shown represent the proportion of plotted events within each gate. Gating analysis was performed in FlowJo v10.10.0*

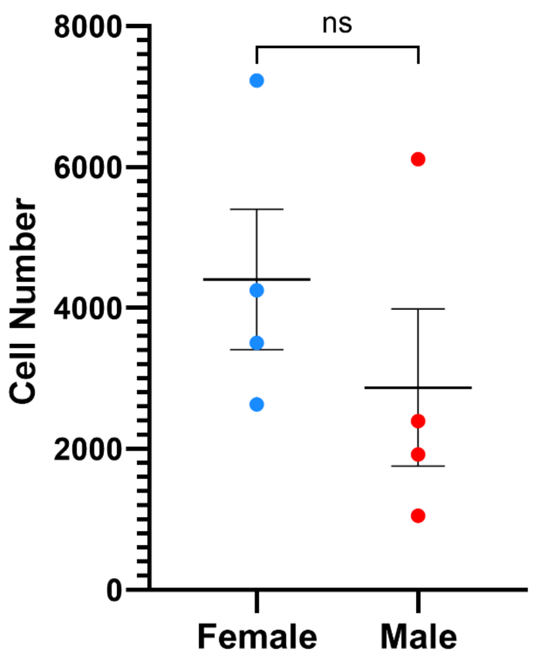

Figure S6. Sex effects in $CD45^+$ cells in AAV2.CAG.RHO.GFP treated wild-type mice. *$CD45^+$ cells detected in AAV treated retina from the wild-type cohort in the Rho^{P23H} AAV response experiment split by subject sex. Similar analysis was not possible in the Rho^{P23H} cohort due to a more skewed ratio between sexes.*

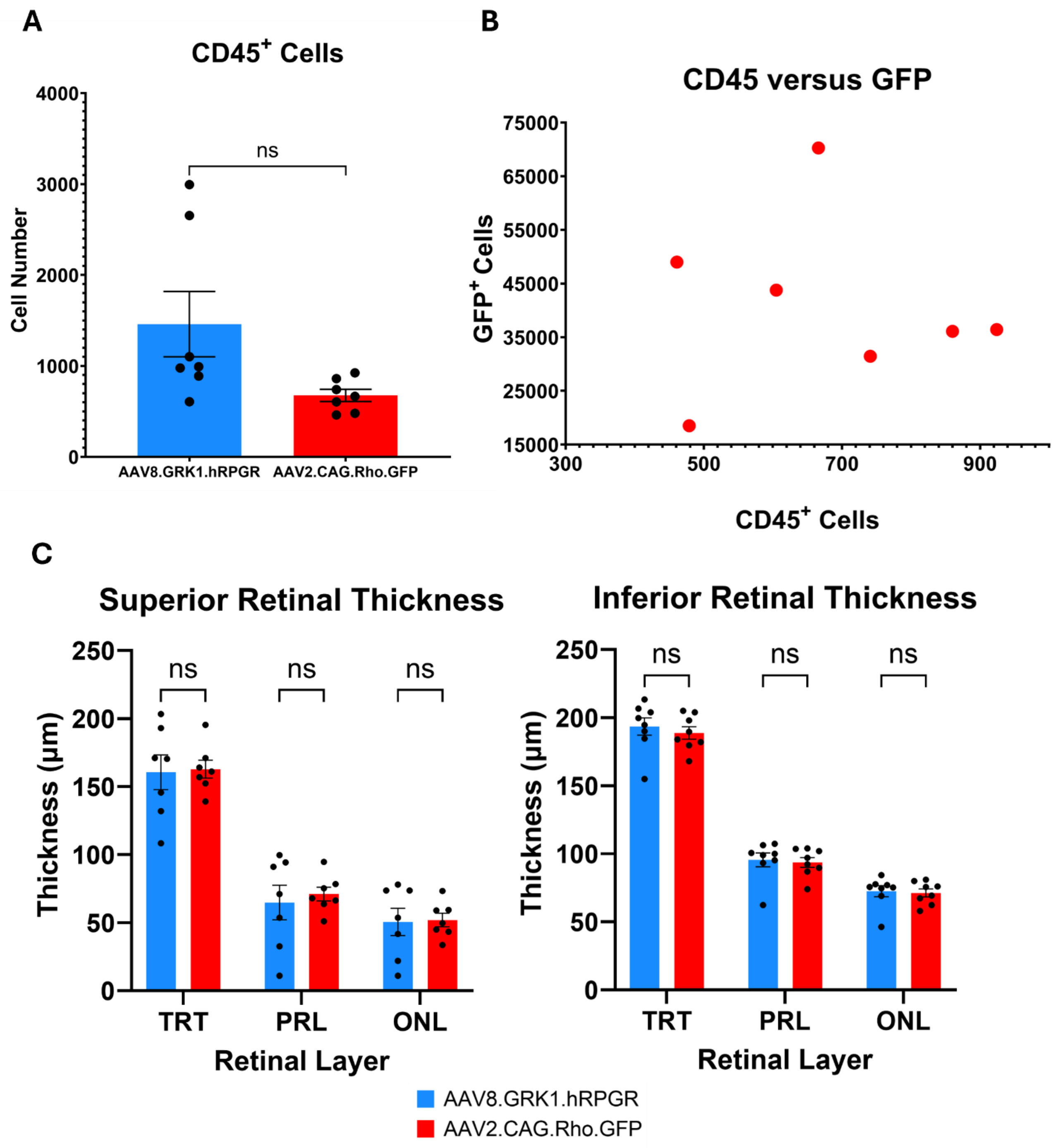

Figure S7. Paired eye comparison of vectors. *9-week-old wild type mice (n=7) received paired eye injections of 1.5x109gc of each vector, with immune infiltration assessed by SLO/OCT imaging at 3 weeks post injection, and eyes harvested for multicolour flow cytometry analysis at 3 weeks post injection. No significant difference was seen in total CD45+ cell populations in treated retinas between the two vectors (p= 0.0625, Wilcoxon matched-pairs signed rank test). CD45+ cells additionally did not correlate with GFP+ cells in AAV2.CAG.RHO.GFP treated retina. There was no significant difference seen in retinal thickness across layers between different vector-treated eyes (Total retinal thickness (TRT), Photoreceptor layer (PRL), Outer nuclear layer (ONL)).*
