# Supplementary material for: Pre-existing retinal inflammation exacerbates gene therapy-associated uveitis in more rapidly progressive retinal degeneration
Source: Mol Ther Adv. 2025 Dec 26;34(1):201652. doi: 10.1016/j.omta.2025.201652 (PMC13370167; doi:10.1016/j.omta.2025.201652)
Supplement: Document S2. Article plus supplemental information [file mmc2.pdf]

Molecular Therapy
Advances
Original Article

Pre-existing retinal inflammation exacerbates gene therapy-associated uveitis in more rapidly progressive retinal degeneration

Molly C. John,[1] Cristina Martinez-Fernandez de la Camara,[1,2] Ahmed Salman,[1] Federica Staurenghi,[1] Hoda Shamsnajafabadi,[1] Michelle E. McClements,[1] Jasmina Cehajic-Kapetanovic,[1,2] Alissa Bray,[3] M Dominik Fischer,[1,2] Andrew D. Dick,[4,5,6] Robert E. MacLaren,[1,2] and Kanmin Xue[1,2,7]

[1]Nuffield Laboratory of Ophthalmology, Nuffield Department of Clinical Neurosciences, University of Oxford, Oxford, UK; [2]Oxford University Hospitals NHS Foundation Trust, Oxford, UK; [3]Oxford Biomedica PLC, Oxford, UK; [4]Academic Unit of Ophthalmology, University of Bristol, Bristol, UK; [5]NIHR Biomedical Research Centre of Ophthalmology, Moorfields Eye Hospital, London, UK; [6]UCL Institute of Ophthalmology, London, UK; [7]Great Ormond Street Hospital for Children NHS Foundation Trust, London, UK

Inherited retinal diseases (IRDs) are often associated with signs of low-grade intraocular inflammation. With gene therapy-associated uveitis (GTAU) being recognized as a critical determinant of the efficacy of retinal gene therapies for IRDs, it is unclear whether such background inflammation might compound any immune response to viral vectors. We explored the immunological state of the retinas of *Rpgr*$^{-/y}$ mice as a model of slow-progressive retinitis pigmentosa (RP) and Rho^{P23H} mice as a model of rapid-progressive RP utilizing multicolor flow cytometry. We subsequently assessed the cellular immune response to subretinal adeno-associated virus (AAV) gene therapy in these mouse models versus age-matched wild-type controls. Rho^{P23H} mice exhibit increased immune cells within the retina, suggesting a degree of blood-retinal barrier breakdown, while *Rpgr*$^{-/y}$ retina remained immunologically quiescent. Subretinal gene augmentation therapy with clinically relevant AAV vectors resulted in a Th1 cell-mediated immune response in both models, but significantly greater immune cell infiltration was seen in Rho^{P23H} retinas while the response in *Rpgr*$^{-/y}$ mirrored that of wild-type controls. Our findings indicate that background immunological changes in rapid retinal degeneration could compound the immune response to gene therapy, thus leading to clinically significant GTAU.

INTRODUCTION

Inherited retinal diseases (IRDs) are a heterogenous group of diseases characterized by progressive degeneration of photoreceptors and irreversible vision loss. Over 300 genes have been implicated in IRDs (https://retnet.org/summaries#a-genes), which encompass all modes of genetic inheritance and variable rates of natural disease progression. Voretigene neparvovec (Luxturna, Spark Therapeutics Inc., Philadelphia, USA) is the first gene therapy to gain regulatory approval for the treatment of *RPE65*-associated Leber congenital amaurosis (LCA) with over 150 patients having received treatment either as part of clinical trials or post-approval globally (Fischer et al.[1]; Russell et al.[2]). Numerous other adeno-associated virus (AAV)-based approaches for IRDs are in advanced stages of clinical development with >100 registered clinical trials.[2–8] AAV-mediated gene therapies are also under evaluation for the treatment of more common forms of retinal degeneration such as age-related macular degeneration (AMD).[9] Despite rapid progress in the field, clinical trials and real-world patient data indicate that intraocular immune and inflammatory response to retinal gene therapy, termed gene therapy-associated uveitis (GTAU), constitute a major determining factor for the safety, efficacy, and durability of all AAV-mediated retinal gene therapies. GTAU has been noted to manifest with a range of clinical features, including vitritis, subretinal infiltrates, choroidal thickening, optic disc swelling, and chorioretinal atrophy.[5,9–13] Post-treatment inflammation can lead to reduced photoreceptor function or cell loss, resulting in reduced visual function that could negate treatment benefits. This highlights the need for a greater understanding of the causes of GTAU to minimize its incidence and severity.

Intraocular inflammation is a known component of several inherited and non-inherited retinal diseases, including retinitis pigmentosa (RP) and age-related macular degeneration, and its severity could influence the rate of disease progression.[14–17] Studies have highlighted immune cell infiltration into the retinas of IRD patients and animal models, as well as the detection of pro-inflammatory cytokines (e.g., TNF-α, IL-6, IL1β, MCP-1, and MCP-2) in the eye.[18–21] Consequently, the degenerating retina has an altered immunological state in comparison to healthy retinas, which are considered immune privileged due to the presence of the blood-retinal barrier (BRB)

Received 8 October 2025; accepted 18 December 2025;
https://doi.org/10.1016/j.omta.2025.201652.

Correspondence: Kanmin Xue, Nuffield Department of Clinical Neurosciences, University of Oxford, Level 6 West Wing, John Radcliffe Hospital, Headley Way, Oxford OX3 9DU, UK.
E-mail: kanmin.xue@eye.ox.ac.uk

Published by Elsevier Inc. on behalf of The American Society of Gene and Cell Therapy.
This is an open access article under the CC BY license (http://creativecommons.org/licenses/by/4.0/).

and local release of immunomodulatory factors.[22,23] Delivery of subretinal AAV has also resulted in different clinical immunological outcomes between disease conditions, even in cases where the vector itself is unchanged. The AAV2.7m8-aflibercept vector (ADVM-022) has been used in trials for both diabetic macular edema and neovascular AMD (INFINITY NCT04418427 and OPTIC NCT03748784, respectively). Despite delivery of the same AAV dose, adverse outcomes were wildly different, with minimal steroid-responsive inflammation reported in OPTIC compared to four cases of severe panuveitis leading to a halt in the INFINITY trial.[24,25] Diabetic macular edema characteristically involves a greater degree of inflammation and BRB breakdown.[26] Additionally, meta-analysis of retinal gene therapy trials indicates an overall prevalence of GTAU of 0.21, though with significant heterogeneity between conditions.[27] We hypothesize that preexisting retinal inflammation may alter retinal immune response to viral vector-mediated subretinal gene therapy in IRD patients, thus leading to an increased risk of GTAU.

Previous studies characterizing the nature and extent of retinal immune responses to AAV have primarily been undertaken in wild-type healthy retinas.[28] A greater understanding of the relationship between disease etiology or the stage of IRD progression and the risk of GTAU could help reduce the incidence and severity of gene therapy-induced ocular adverse events. In this study, we investigated two IRD models: a slow-progressing mouse model of X-linked RP (*Rpgr*$^{-/y}$)[29] and a rapidly progressive murine Rho^{P23H} model. The *Rpgr*$^{-/y}$ mouse is a knockout of the mouse *Rpgr* gene via disruption of exons 4–6, which results in loss of *Rpgr* expression and slowly progressive retinitis degeneration. This phenotype has previously been characterized in relation to retinal health and function.[29] In brief, Rpgr$^{-/y}$ mice retain normal retinal architecture without significant photoreceptor loss up to 6 months of age, suffering subsequent outer-nuclear layer thinning and notable photoreceptor degeneration corresponding to minor reductions in electroretinography (ERG) a-wave amplitudes. Patients with *RPGR*-associated X-linked RP harbor a wide range of genetic mutations in the *RPGR* gene, which results in phenotypic heterogeneity and varied speeds of disease progression.[30] The *Rpgr*$^{-/y}$ mouse most closely models a milder phenotype seen in patients with pathogenic mutations in exons 1 to 14 of human *RPGR* as opposed to ORF15 or complete *RPGR* loss.[31,32] The Rho^{P23H} mouse (B6.129S6(Cg)-Rhotm1.1Kpal/J, Jackson Laboratory) is a model of autosomal-dominant RP. It carries a C-to-A nucleotide substitution, resulting in a proline-to-histidine change at amino acid position 23 (P23H) of the endogenous mouse *rhodopsin* gene, which is representative of a mutation hotspot in humans. The heterozygous Rho^{P23H} mouse models the human disease and exhibits a rapidly progressive outer retinal degeneration with photoreceptor loss and degeneration evident by postnatal day 35. In this study, we investigated the background immunological state of the retinas in both models and their differential response to subretinal gene therapy with relevant AAV vectors using *in vivo* retinal imaging, multiparameter flow cytometric analysis, and immunohistochemistry with a range of immune and cell death markers.

RESULTS

Phenotype of slow and rapid mouse models of inherited retinal degenerations

Rpgr$^{-/y}$ *mouse with slowly progressive X-linked RP*

Affected male *Rpgr*$^{-/y}$ mice were assessed to confirm the anticipated RP phenotype via confocal scanning laser ophthalmoscopy (cSLO) and optical coherence tomography (OCT) (Heidelberg Spectralis, Heidelberg Engineering, Heidelberg, Germany). Retinal layer morphology and thickness were compared to those of age and sex-matched wild-type (C57BL6/J) controls. cSLO revealed the appearance of hyperreflective foci across the retina of *Rpgr*$^{-/y}$ mice, which increased from 3 to 12 months of age (Figure 1A). These hyperreflective spots did not appear to correlate with any retinal or retinal pigment epithelium (RPE) structural changes on the corresponding OCT. Retinal thickness measurements from radial OCT line scans superiorly and inferiorly showed slowly progressive retinal thinning in *Rpgr*$^{-/y}$ mice from 3 to 6 months of age (Figure S1B). While significant retinal thinning can be seen in 12-month-old *Rpgr*$^{-/y}$ mice compared with age-matched controls (21.9% decrease in total retinal thickness, two-way ANOVA $p \leq 0.0001$), preserved outer retinal layers suggest surviving photoreceptors (Figures 1B and 1C). The degenerative changes correlated with mild reduction in rod responses as determined by ERG, indicating incomplete photoreceptor loss (Figure S1C).

The presence of functional photoreceptors and ERG response in 12-month-old *Rpgr*$^{-/y}$ mice would suggest that, despite a degree of degeneration, the retina may be amenable to rescue by gene augmentation therapy targeting the surviving photoreceptors. Therefore, these aged *Rpgr*$^{-/y}$ mice serve as a model for patients undergoing retinal gene therapy for X-linked RP associated with *RPGR* mutations. They were used to assess the background immunological state of the retina and the immune response to subretinal AAV-mediated gene therapy.

Rho(P23H) mouse with rapidly progressive RP

Rho^{P23H} mice were assessed for the retinal phenotype at 9 weeks of age as this was determined to represent a late—but not complete—stage of retinal degeneration. cSLO imaging revealed hyperreflective foci in the 9-week-old Rho^{P23H} retina that were absent in age-matched controls (Figure 2A). These foci were evenly distributed across the retina, though less numerous than those seen in the *Rpgr*$^{-/y}$ retina. Similarly, they did not correlate with any noticeable features on the OCT. OCT demonstrates clear retinal thinning in the Rho^{P23H} retinas although there was preservation of retinal lamination (Figure 2B). Retinal thickness measurements obtained from OCT showed significant thinning in Rho^{P23H} (n = 8) versus age-matched controls (n = 8) across the outer retinal layers, indicative of photoreceptor loss (mean photoreceptor layer thickness 52.6 μm versus 100.9 μm, p < 0.0001, two-way ANOVA).

While the photoreceptor layer is significantly reduced compared with wild-type retinas, OCT analysis confirmed that the Rho^{P23H} mouse retained photoreceptors up to 9 weeks. This time point was subsequently chosen for the analysis of immune cell populations

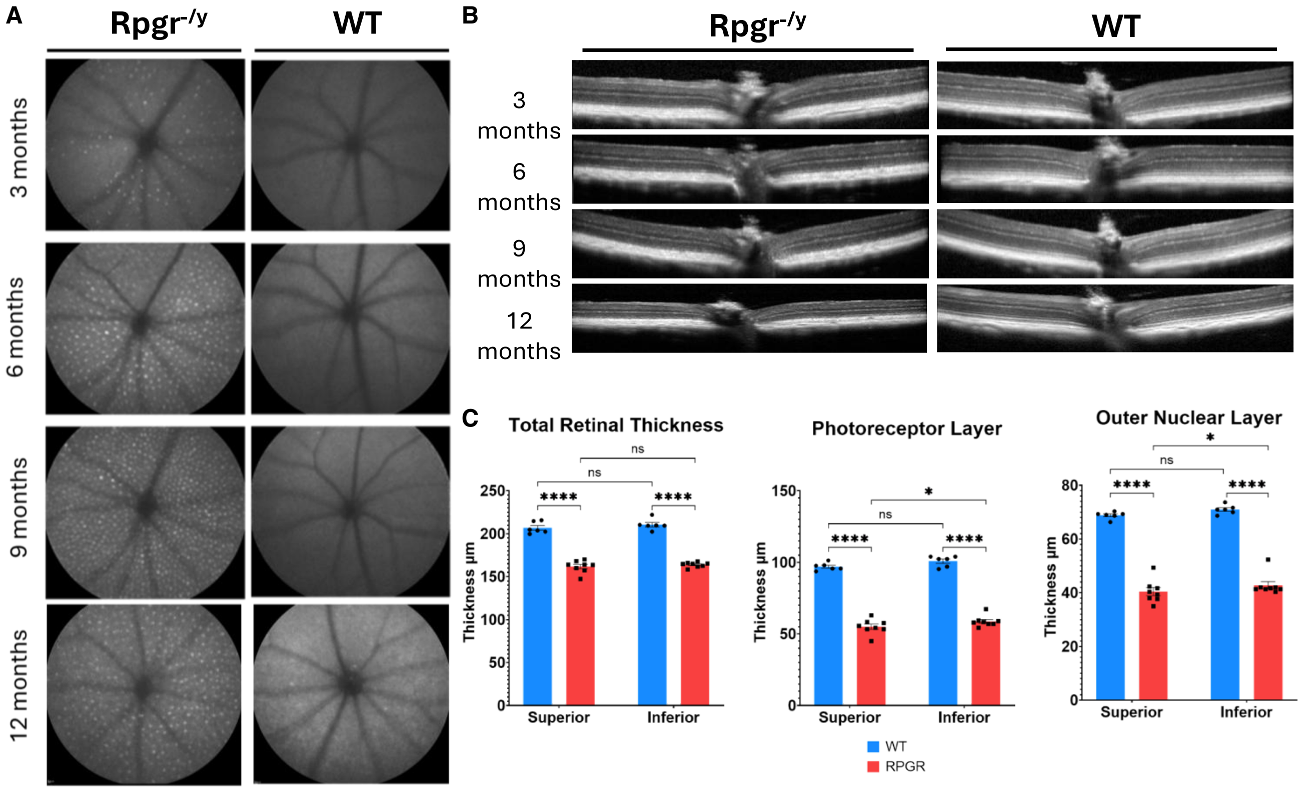

Figure 1. Slow progressive retinal degeneration in *Rpgr*$^{-/y}$ mice

(A) Confocal scanning laser ophthalmoscopy (cSLO) of *Rpgr*$^{-/y}$ mice and age-matched wild-type (C57BL6/J) controls. Representative images showing increasing hyperreflective spots across the whole retina in *Rpgr*$^{-/y}$ from 3 months of age. (B) These hyperreflective spots do not appear to correlate with any noticeable structural changes on optical coherence tomography (OCT) in 12-month-old *Rpgr*$^{-/y}$ mice, while moderate retinal thinning was seen compared with age-matched controls. (C) Quantification of retinal thickness changes in 12-month-old *Rpgr*$^{-/y}$ mice (n = 6) demonstrating outer retinal thinning in both superior and inferior retina compared with age-matched controls (n = 6). Error bars represent standard errors of the mean (*SEM*). ****p < 0.0001 two-way ANOVA.

during active degeneration, rather than at a "burnt out" end stage. The presence of viable photoreceptors similarly indicates that this time point can be considered to model a disease stage in which a patient would still be considered amenable to gene therapy, and therefore, analysis is clinically relevant.

Background immunological state of retinas in IRD models

Aged Rpgr$^{-/y}$ *mouse retinas do not show significant immune cell infiltration*

To determine the background immunological state of the retina in aged *Rpgr*$^{-/y}$ mice, retinas from 12-month-old affected males (n = 8 retina) and age-matched controls (n = 4) were dissociated for multicolor flow cytometry analysis using an optimized panel of leukocyte markers (Figure S2A).[28] Immune cells were identified by the pan immune cell marker CD45 and sub-gated for additional leukocyte markers. Microglia and macrophages were distinguished by their relative CD45 and CD11b expression; microglia were classified as CD45^{Lo}:CD11b^{Hi}, whereas macrophages classified as CD45^{Hi}:CD11b^{Hi}. The overall retinal immune cell numbers did not significantly differ between the *Rpgr*$^{-/y}$ and wild-type retinas (mean of 0.42% vs. 0.46% of live cells, p = 0.8682). These are consistent with previous data on retinal leukocyte populations in healthy murine retinas.[28] In both groups, the majority of immune cells identified were CD45^{lo}CD11b^{hi}, and thus likely represent the resident microglia population. Interestingly, this population was significantly enriched in *Rpgr*$^{-/y}$ retinas (p = 0.0471) despite the lack of overall immune cell change. Minor populations of CD45^{hi}CD11b^{hi} macrophages and CD19^{+} B cells were found in both groups, though in all cases the cell numbers were limited and no differences were seen between *Rpgr*$^{-/y}$ and wild-type retinas. Further sub-gating on additional leukocyte markers was not possible due to the low cell numbers, with populations of CD4 and NK1.1 cells not distinguishable from background. The lack of other identifiable populations and overall low number of blood-derived immune cells suggest a lack of immune cell infiltration in the *Rpgr*$^{-/y}$ retina and limited BRB breakdown.

To further interrogate the immune environment, immunohistochemistry (IHC) was performed on intact retinas. Staining in retinal sections from 12-month-old *Rpgr*$^{-/y}$ retinas showed minimal CD45

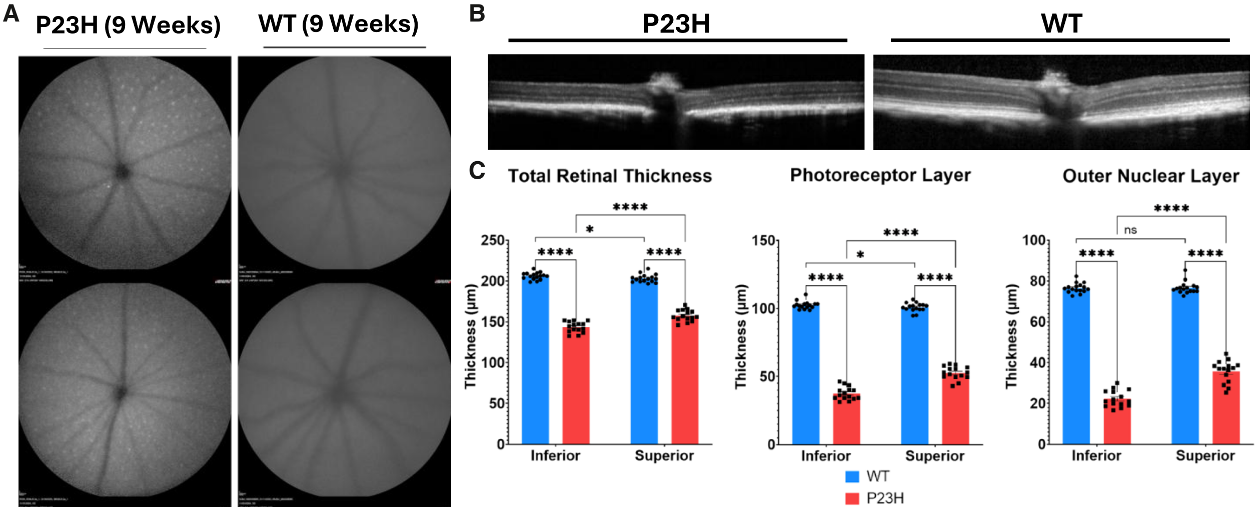

Figure 2. Rapid outer retinal degeneration in Rho(P23H) mice

(A) SLO comparison of 9-week-old Rho(P23H) mice versus age-matched wild-type (C57BL6/J) controls. Hyperreflective spots are visible on Rho(P23H) SLO. (B) OCT comparison of Rho(P23H) versus age-matched controls. (C) Quantification of retinal thickness at 9 weeks of age in Rho(P23H) (n = 8) versus control (n = 8) mice. ****p < 0.0001 two-way ANOVA.

positivity, overlaying mild Iba1 expression in the inner retina in both wild-type and *Rpgr*$^{-/y}$ sections, likely representing resident microglia (Figure 3B). No observable difference was seen between *Rpgr*$^{-/y}$ and wild-type controls in terms of Iba1-positive cell morphology in retinal sections (Figure 3B).[33]

Lack of significant additional CD45 positivity and CD68 staining was consistent with the lack of significant immune cell infiltration by flow cytometry. Müller glia activation has been reported in a number of IRDs as part of reactive gliosis induced by photoreceptor degeneration.[34] Staining for the marker of Müller glia activation, glial fibrillary acidic protein (GFAP), was performed on retinal sections from 12-month-old *Rpgr*$^{-/y}$ and age-matched wild-type retinas (Figure 3C). No clear differences were seen in the level of GFAP staining between the two groups, thus suggesting a lack of Müller glial involvement in the degenerative phenotype.

To understand the increase in the microglia population noted in flow cytometric analysis, Iba1-stained retinal flat mounts were optically sectioned via confocal microscopy to enable comparison of microglia morphology across individual retinal layers (Figure 3D). At the 1-month time point, *Rpgr*$^{-/y}$ mice exhibited reduced Iba1 staining across all retinal layers compared with wild-type controls. At both 3-month and 6-month time points, no noteworthy differences were observed in overall microglia numbers or staining intensity, except for more intense Iba1 staining in the outer nuclear layer (ONL) of *Rpgr*$^{-/y}$ mice at 3 and 6 months of age. This increased outer retinal staining may indicate microglia migration from the inner retina (ganglion cell layer and inner plexiform layer) toward the photoreceptor layer or subretinal space in response to degenerating photoreceptors. Microglia migration has been noted via Iba1 staining in other models of IRDs.[35] Despite migration to the ONL in *Rpgr*$^{-/y}$ mice, microglia retained a more ramified morphology normally associated with a homeostatic state.

Rapidly degenerating Rho(P23H) retinas demonstrate immune cell infiltration

To determine the background immunological state of the retina in a rapidly degenerating IRD model, retinas from 9- to 10-week-old Rho^{P23H} mice (n = 8 mice) were dissociated for flow cytometric analysis alongside age-matched wild-type (C57BL6/J) controls (n = 8 mice) (Figure S2B). Populations were resolved as in the previous experiment, with the exception of inclusion of a CD8 antibody to aid T cell identification. There were significant differences in immune cell populations between the two groups. Rho^{P23H} retinas contained significantly higher numbers of CD45^{+} immune cells compared with wild-type, representing 16.96% versus 0.91% of live cells, respectively (p < 0.0001) (Figure 4A). Notably, a sex-associated difference in immune cell infiltration among the P23H retinas was also observed. Female P23H mice (n = 4) exhibited a significantly higher number of total CD45^{+} cells compared with male siblings (n = 4) within the cohort, at 1,148 versus 933 mean CD45^{+} cells per retina (p = 0.0395) (Figure 4B). The majority of CD45^{+} cells identified in the retinas were monocytic phagocytes (either microglia or macrophages), which were significantly elevated in Rho^{P23H}. Minor populations of both CD8 and CD4 T cells were seen, which appeared slightly increased in the Rho^{P23H} group, though cell numbers of these populations were very low and fell within background levels. Overall, the findings were indicative of a degree of BRB breakdown with significant leukocyte infiltration, which may prime the retina for gene therapy-associated inflammation.

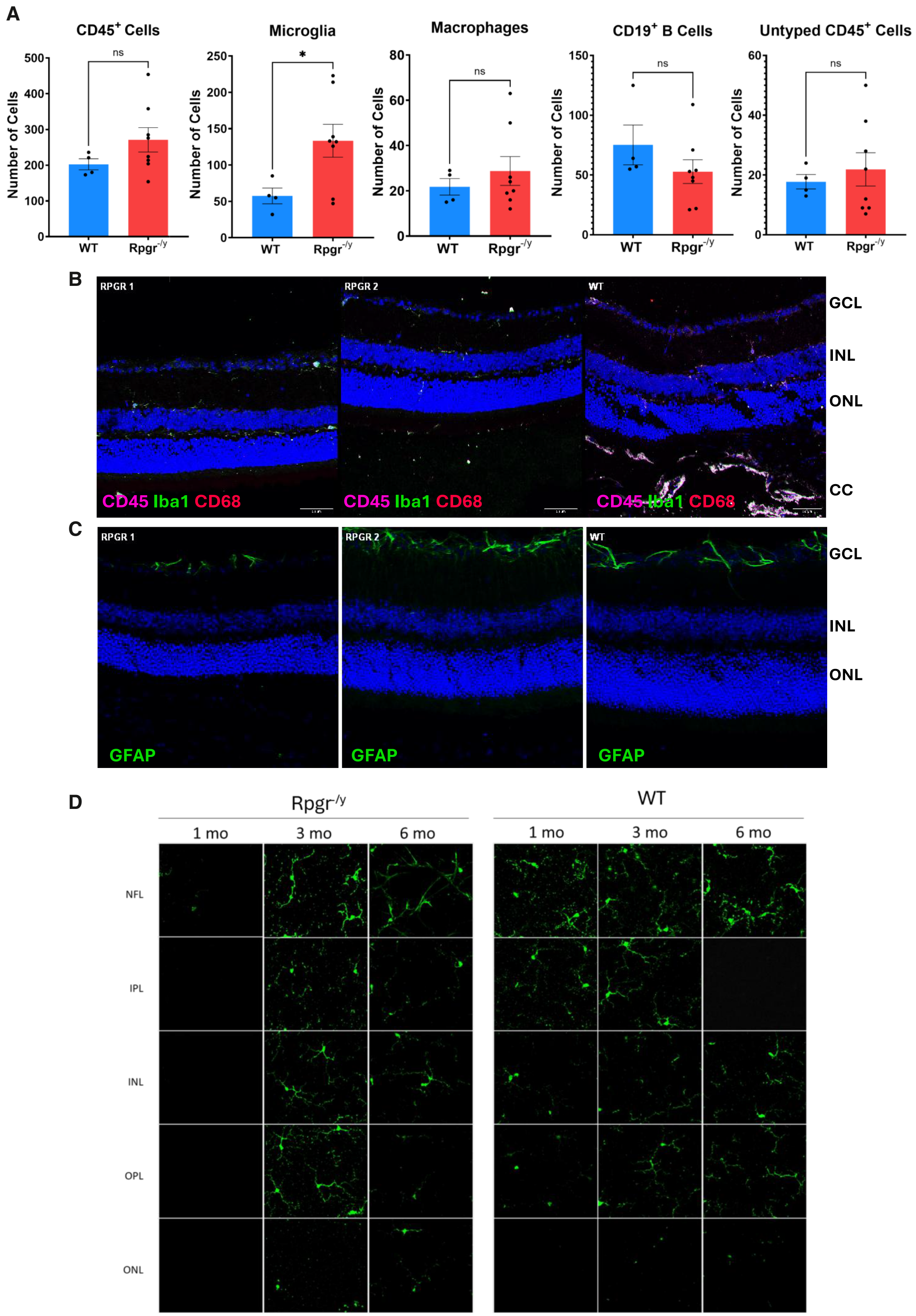

(legend on next page)

Immunostaining was performed as previously for a number of immune markers to validate the flow cytometry findings. GFAP staining appeared increased in 9- to 10-week-old P23H retinas compared with controls (Figure 4C), indicative of Müller glia activation. Microglial migration was evident from increased Iba1-positive cells seen in the ONL of P23H retinal sections along with increased overall Iba1 positivity compared with controls (Figure 4E). Minor CD68 positivity was also seen in these sections, likely corresponding to infiltrating monocytes as detected by flow cytometry (Figure 4F). These results suggest activation of both Müller glia and microglia in the rapidly degenerating P23H retina.

Cell death mechanisms in IRD models

We hypothesized that the more rapid rate of retinal degeneration in Rho^{P23H} compared with $Rpgr^{-/y}$ may be associated with an overwhelming amount of photoreceptor cell death, which could lead to damage-associated molecular pattern (DAMP) signaling, Müller glia/microglia activation, and subsequent immune cell infiltration. The extent of cell death in both models was assessed by TUNEL staining, which identifies apoptotic cells within retinal sections. The sections from 3- to 4-week-old Rho^{P23H} mice showed extensive TUNEL staining in the ONL compared with wild-type controls (Figure 5A), indicative of photoreceptor apoptosis. Some TUNEL-positive cells were also present in the RPE. Retinal sections from 10- to 12-week-old Rho^{P23H} mice were also assessed (Figure S3A). These demonstrate reduced TUNEL positivity but markedly reduced ONL thickness, suggesting that the majority of photoreceptor death had already occurred by this later time point. HGMB1 staining was also assessed as a marker of necrosis, but no clear difference was seen between Rho^{P23H} and wild types (Figure S3B). In contrast, TUNEL staining of retinal sections from 12-month-old $Rpgr^{-/y}$ mice showed minimal to no staining (Figure 5B), indicative of a low rate of apoptotic cell death in this model.

BRB integrity in Rho^{P23H} mice

The presence of infiltrating immune cell populations within the 9-week-old Rho^{P23H} prompted examination of the outer BRB in these mice via ZO-1 staining. RPE flat mounts were taken from 8- to 9-week-old Rho^{P23H} (n = 4) and wild-type (n = 4) mice. ZO-1 staining was evident in both models, with clear hexagonal RPE morphology (Figures 6A and 6B). There were no clear significant discontinuities in Rho^{P23H} that can be considered indicative of large-scale BRB breakdown. Images were obtained at regular intervals on flat mount RPE samples, and ZO-1-positive staining area was quantified across image fields with ImageJ. Quantified staining suggests a trend toward ZO-1 reduction in Rho^{P23H}, though no significant difference was seen (Figure 6C).

Cellular immune response to subretinal AAV-mediated gene therapy in *$Rpgr^{-/y}$* mice mirrors the wild-type response

To assess the immune response to AAV-mediated gene therapy in the slow degenerating model of RP, a cohort of 12-month-old affected male $Rpgr^{-/y}$ mice (n = 5) and age- and sex-matched wild-type (C57BL6/J) controls (n = 5) were treated with subretinal injections of 1.5×10^9 vg of an AAV gene therapy vector (AAV8.GRK1.h*RPGR*) and phosphate-buffered saline (PBS) vehicle control in paired eyes. The AAV vector represents a clinically relevant gene therapy for the treatment of *RPGR*-related X-linked RP (XLRP), which expresses the full-length, codon-optimized human $RPGR^{ORF15}$ coding sequence under the control of GRK1, a photoreceptor-specific promoter.[36,37] At 3 weeks post treatment, the eyes were harvested and neuroretinas (without RPE or choroid) dissociated for multicolor flow cytometry. To ensure that the immune response seen was not due to general retinal toxicity from the AAV treatment, retinal thickness measurements at baseline and post-treatment were compared between AAV and sham-treated eyes by OCT. This showed no significant differences in retinal thickness (Figure 7B), as well as the absence of vitritis or subretinal infiltrates (Figure 7C). Moreover, no significant retinal reflectance changes were seen by cSLO between AAV and sham-treated eyes.

Despite the absence of clinical features of GTAU, both $Rpgr^{-/y}$ and wild-type mice demonstrated an increase in retinal immune cells in the AAV-treated eyes. $CD45^+$ immune cells represented 31.2% and 25.3% of live cells in AAV-treated $Rpgr^{-/y}$ and wild-type eyes, respectively, compared with only 2.1% and 1.7% in PBS-treated eyes (Figure 7D). Consistent with previous data, the majority of $CD45^+$ cells in sham-treated retinas were $CD45^{Lo}$:$CD11b^{Hi}$, which is indicative of microglia. On the other hand, in AAV-treated eyes, subpopulation analysis (Figure S4) identified primarily macrophage ($CD45^{hi}$:$CD11b^{hi}$) and T cell infiltration. Minor populations of $CD19^+$ B cells and $CD11c^+$ dendritic cells were also identified. Two unknown populations were isolated in AAV-treated eyes: one positive for only CD3 and CD45 (noted as "other $CD3^+$") and another negative for all markers except CD45 (unclassified CD45 cells). $CD3^+$-only cells may constitute naive T cells, while the latter may represent precursor immune cells or cells that have lost their surface markers during tissue processing. Across all the AAV-treated retinas, there was no significant difference between the nature and magnitude of immune responses seen in $Rpgr^{-/y}$ and wild-type mice.

Rho(P23H) mice exhibit increased cellular immune responses to subretinal gene therapy

In an analogous experiment, 9-week-old Rho^{P23H} mice (n = 8) and age-matched wild-type controls (n = 8) were treated with subretinal

Figure 3. Aged *$Rpgr^{-/y}$* mouse retinas show microglia migration but no significant immune cell infiltration

(A) Multicolor spectral flow cytometry of dissociated $Rpgr^{-/y}$ retinas (n = 8) versus control retinas (n = 4). (B) CD45, Iba1, and CD68 immunostaining did not detect any significant infiltrating immune cells in the inner or outer retina of $Rpgr^{-/y}$ mice, comparable to age-matched wild-type control (C57BL6/J) retinas (n = 4 eyes stained per genotype). (C) Immunostaining for GFAP did not show any significant difference between $Rpgr^{-/y}$ and control retinas. (D) Retinal flat mount staining for Iba1 showing migration of microglia in $Rpgr^{-/y}$ to the outer nuclear layer (ONL) in 3- and 6-month-old mice. Nerve fiber layer (NFL), inner plexiform layer (IPL), inner nuclear layer (INL), outer plexiform layer (OPL), ONL. *$p < 0.05$ (two-tailed unpaired t test).

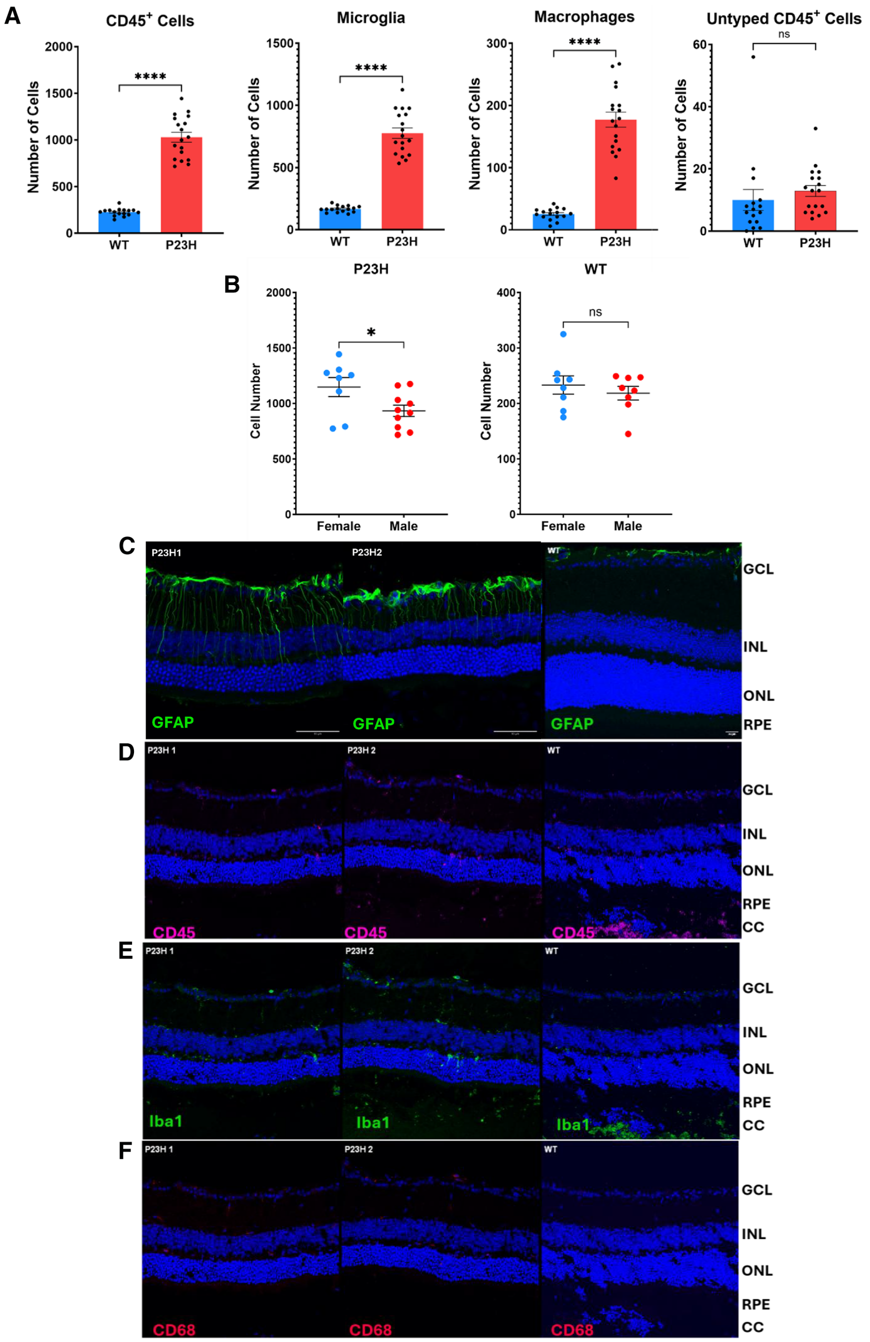

Figure 4. Nine-week-old Rho(P23H) mouse retinas show immune cell infiltration consistent with BRB breakdown

(A) Multicolor flow cytometric analysis of immune cell populations within dissociated 9- to 10-week-old Rho^{P23H} retinas (n = 8 mice) versus age-matched WTs (n = 8 mice). Each data point represents one retina. ****$p < 0.0001$, **$p < 0.001$ (two-tailed unpaired t test). (B) $CD45^+$ cell numbers by sex in the Rho^{P23H} versus WT mice showed increased immune infiltration in female IRD mice compared to males (n = 8 retinae per group). (C) GFAP staining of 9- to 10-week-old P23H retinal sections shows increased GFAP expression versus age-matched WT C57BL6/J. (D) CD45 staining as a pan immune cell marker showed immune cells within the Rho^{P23H} retina. (E) Iba1 expression and microglial migration to the ONL. (F) Minor CD68 staining was seen in the Rho^{P23H} retina. (D–F) Represent individual channels from co-stained sections (n = 4 eyes stained per genotype).

injections of 1.5×10^9 vg of an AAV vector (AAV2.CAG.*RHO*.eGFP) and PBS sham in paired eyes. In this case, the AAV vector expressed human rhodopsin tagged with enhanced green fluorescent protein (GFP) under the control of a ubiquitous chicken beta actin (CAG) promoter. Eyes were harvested at 3 weeks post treatment and retinas dissociated for flow cytometric analysis (Figure 8A). No significant difference in retinal thickness was seen between AAV- and sham-treated eyes of either Rho^{P23H} or wild-type mice (Figure 8B).

In vivo retinal imaging by cSLO and OCT demonstrated low-level of vitritis in Rho^{P23H} AAV-treated eyes, as well as evidence of subretinal infiltrates (Figure 8C) but not in the sham-treated or wild-type eyes. Increased infrared reflectance in the superior retina of AAV-treated eyes likely represents GFP-tagged transgene expression, which correlates with the superior approach of the subretinal injections. GFP expression was not significantly different between AAV-treated eyes of Rho^{P23H} and wild-type mice, with GFP^+ cells accounting for 52.53% and 57.03% of live cells, respectively (p = 0.4278). Sham-injected eyes appeared normal by retinal imaging.

Flow cytometric analysis showed a significant increase in $CD45^+$ immune cells in AAV-treated eyes compared with PBS-treated controls in both mouse strains (wild type [WT] p = 0.001, Rho^{P23H} $p \leq 0.0001$) (Figure 8D). Greater $CD45^+$ cell numbers in sham-treated P23H eyes compared to wild type correlated with the background difference seen in the previous experiment, though significance was not seen in this experimental setup due to multiple comparison testing. Total immune cell numbers were significantly greater in the AAV-treated Rho^{P23H} eyes versus wild-type eyes (10.3% of live cells versus 5.6%, p = 0.0003). Subpopulation identification (Figure S5) showed a consistent type-I cellular immune response dominated by T cells and macrophages. CD4 T cells represented the majority of T cell population, with approximately double the number compared to CD8 T cells (average 1,855 versus 604 cells in Rho^{P23H} retina). In addition, increased numbers

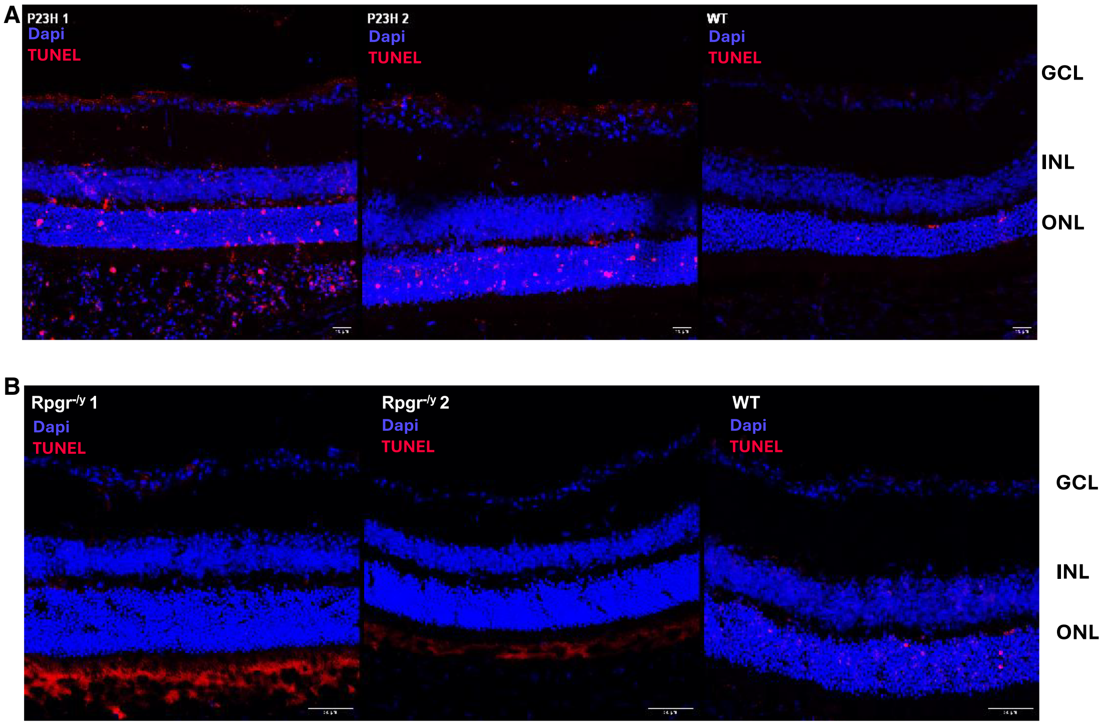

Figure 5. TUNEL staining indicating the extent of cell death in Rho^{P23H} (P23H) and Rpgr$^{-/y}$ retinas

(A) TUNEL staining in two representative 3- to 4-week-old Rho^{P23H} retinas shows extensive outer retina TUNEL positivity. (B) TUNEL staining in two representative 12-month-old *Rpgr*$^{-/y}$ retinas versus wild-type control showed minimal apoptotic cells.

of B cells, dendritic cells, natural killer cells, and the two aforementioned, as-yet not fully phenotyped CD45$^+$ populations were detected in the AAV-treated eyes, although numbers were relatively low. The AAV-treated Rho^{P23H} retinas showed the greatest number of all the major immune cell subgroups, which is consistent with imaging evidence of cellular infiltrates. Despite differences in immune cell populations, expression of the GFP transgene was the same between AAV-treated wild-type and P23H mice (52.53% versus 57.03% of live cells, respectively, p = 0.4278, two-tailed unpaired t test).

DISCUSSION

The subretinal space is considered an immune-privileged space protected by BRBs, which makes it an ideal site for the administration of viral vector-mediated gene therapy for IRDs. Our observations suggest that differing immune responses to AAV gene therapy observed in different IRD patients may be, in part, explained by the varying background immune status of the retinas in these conditions. Patients with a more rapidly progressive IRD associated with increased retinal inflammation may have greater inherent risk of developing gene therapy-associated intraocular inflammation, which could lead to poorer visual outcomes. Our data show an exaggerated cell-mediated immune response in the rapidly degenerating Rho^{P23H} retina, correlated with increased BRB breakdown and immune cell infiltration at baseline. In contrast, the relatively immunologically quiescent Rpgr$^{-/y}$ retina responded similarly to subretinal AAV gene therapy as healthy wild-type eyes.

Rho^{P23H} retina exhibited an increased immune cell presence in the retina at 9–10 weeks of age, consisting primarily of macrophages and increased microglial numbers. Activation of microglia is considered a hallmark across a number of IRDs, with activated microglia exhibiting strongly enhanced proliferation and migration.[38,39] Our flow cytometry data are suggestive of microglia activation, alongside a breakdown in the BRB in Rho^{P23H} mice. Since increased Iba1 staining was seen in the ONL, the increase in microglia numbers may be attributed at least in part to proliferation of resident microglia. In addition, highly elevated macrophage numbers in the presence of increased lymphocyte populations would suggest immune cell infiltration from the peripheral blood and breakdown of the BRB. Further investigation of the BRB via ZO-1 staining suggested a relatively intact outer BRB in the form of RPE tight junctions. Changes to RPE morphology and ZO-1 staining have previously been reported in various models of RP. Notably, these gross discontinuities in ZO-1 staining and changes to the hexagonal array were seen in older mice, e.g., rd9 mice over 1 year of age and rd10 mice following nearly complete rod death. More subtle changes may be present at the earlier time point studied here, though not easily detectable via staining. Retinal vascular leakage and immune cell infiltration into the inner retina may also occur as a consequence of inner BRB disruption,

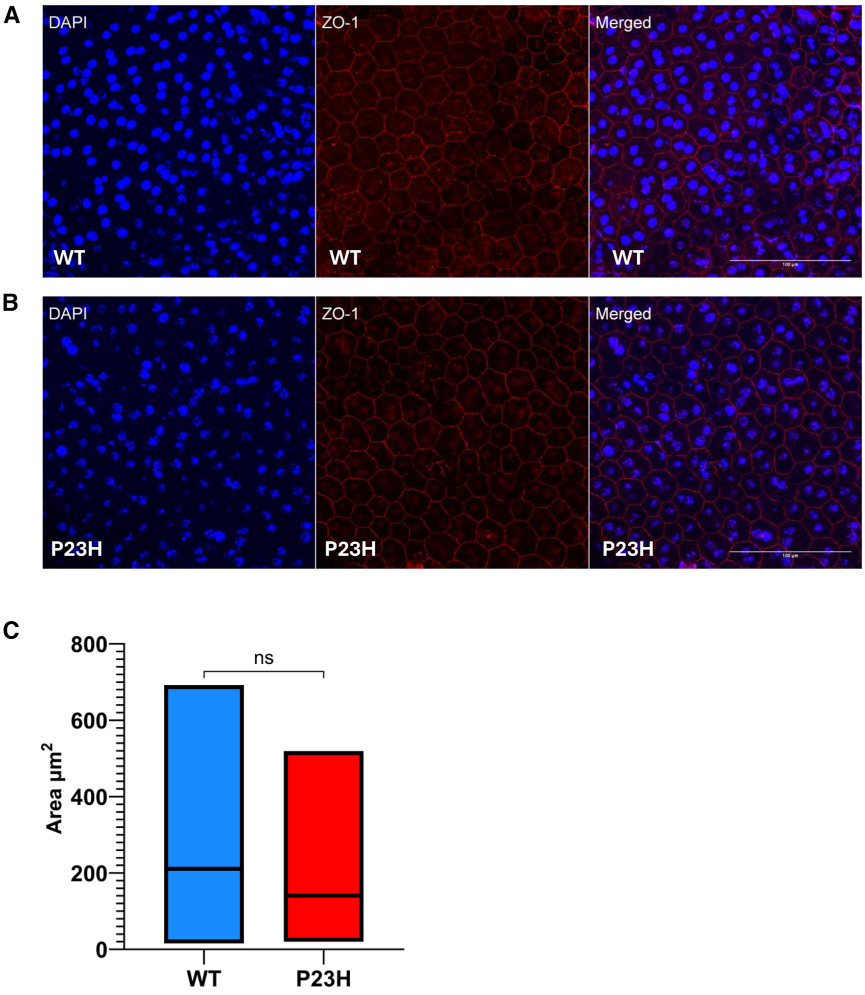

Figure 6. ZO-1 staining shows intact tight junctions in Rho^{P23H} outer BRB

(A) Representative ZO-1 staining of C57BL6/J wild-type RPE flat mount. (B) Representative staining of 9-week-old Rho^{P23H} RPE flat mount. (C) No significant difference was seen in the ZO-1-positive area across image fields between wild-type and Rho^{P23H} flat mounts (n = 4 eyes per genotype, 4 fields per eye). Area was quantified as signal above threshold in ImageJ (p = 0.399, two-tailed unpaired t test).

which would not be detected by ZO-1 staining. Additional investigation via fluorescein angiography in the future may help clarify the integrity of the inner BRB in this rapid retinal degeneration model.

It should also be noted that activated microglia express elevated levels of CD45, potentially clouding the gating strategy used here to distinguish microglia and macrophages in inflamed retina. This may explain part of the increase seen in microglia numbers, as proliferation alone is unlikely to result in the magnitude of increase seen here. Use of additional microglia-specific markers (e.g., TMEM119 and P2RY12) may be considered, although these provide limited utility as their expression is significantly altered upon microglial activation.[40] Lineage-tracing protocols are currently the best method for resolving resident microglia and infiltrating macrophages, and such an approach could be considered in the future.[41,42] Alongside cellular infiltration and microglial activation, generalized inflammatory responses were also seen in Rho^{P23H} retina in the form of Müller glial activation, indicating increased gliosis. Increased GFAP staining has been reported in Rho$^{-/-}$ models alongside other markers of reactive gliosis.[43] Activated Müller glia may serve as a source of cytokines and pro-inflammatory factors that induce BRB breakdown.[43,44]

In contrast, *Rpgr*$^{-/y}$ mouse retinas remained immunologically inactive despite slowly progressive retinal degeneration up to 1 year of age. No significant increase was observed in CD45^{+} immune cells by flow cytometry analysis, and overall cell numbers of all immune populations remained low. The sample size for the flow cytometric quantification of CD45^{+} retinal cells was limited, and smaller yet potentially meaningful shifts in CD45^{+} cell abundance may therefore not have reached statistical significance. Nonetheless, the finding remained consistent across independent experiments. The small increase in microglia observed in *Rpgr*$^{-/y}$ retina may reflect microglia migration to the outer retina where a slow rate of photoreceptor degeneration occurs. The absence of macrophages or lymphoid cells indicates that the BRB remains intact in this model, with a lack of peripheral immune system involvement. ZO-1 staining of the RPE monolayer may serve to demonstrate the intact state of the outer BRB in this model.

Differences in cell death rates between the two IRD models could explain the difference in background immune states seen. TUNEL staining indicated high levels of apoptotic cells within the Rho^{P23H} retina compared to minimal apoptosis in *Rpgr*$^{-/y}$. Clearance of apoptotic photoreceptors via microglial phagocytosis may be overwhelmed in Rho^{P23H} retinas, leading to release of DAMP signals such as HSP70, Aβ, uric acid, heparin sulfate, and other metabolic or nuclear products recognized by innate receptors.[45] Innate signaling could trigger microglia and Müller glia activation, promoting inflammatory cytokine release and immune cell infiltration.[46] Further direct interrogation of DAMP markers may aid in establishing this as a mechanism. In comparison, minimal, slow photoreceptor death in *Rpgr*$^{-/y}$ retinas may remain at a rate within the phagocytic capacity of resident microglia without increased pro-inflammatory signaling. Equally, the prolonged, slow exposure to low-level photoreceptor death-related DAMPs over the extended

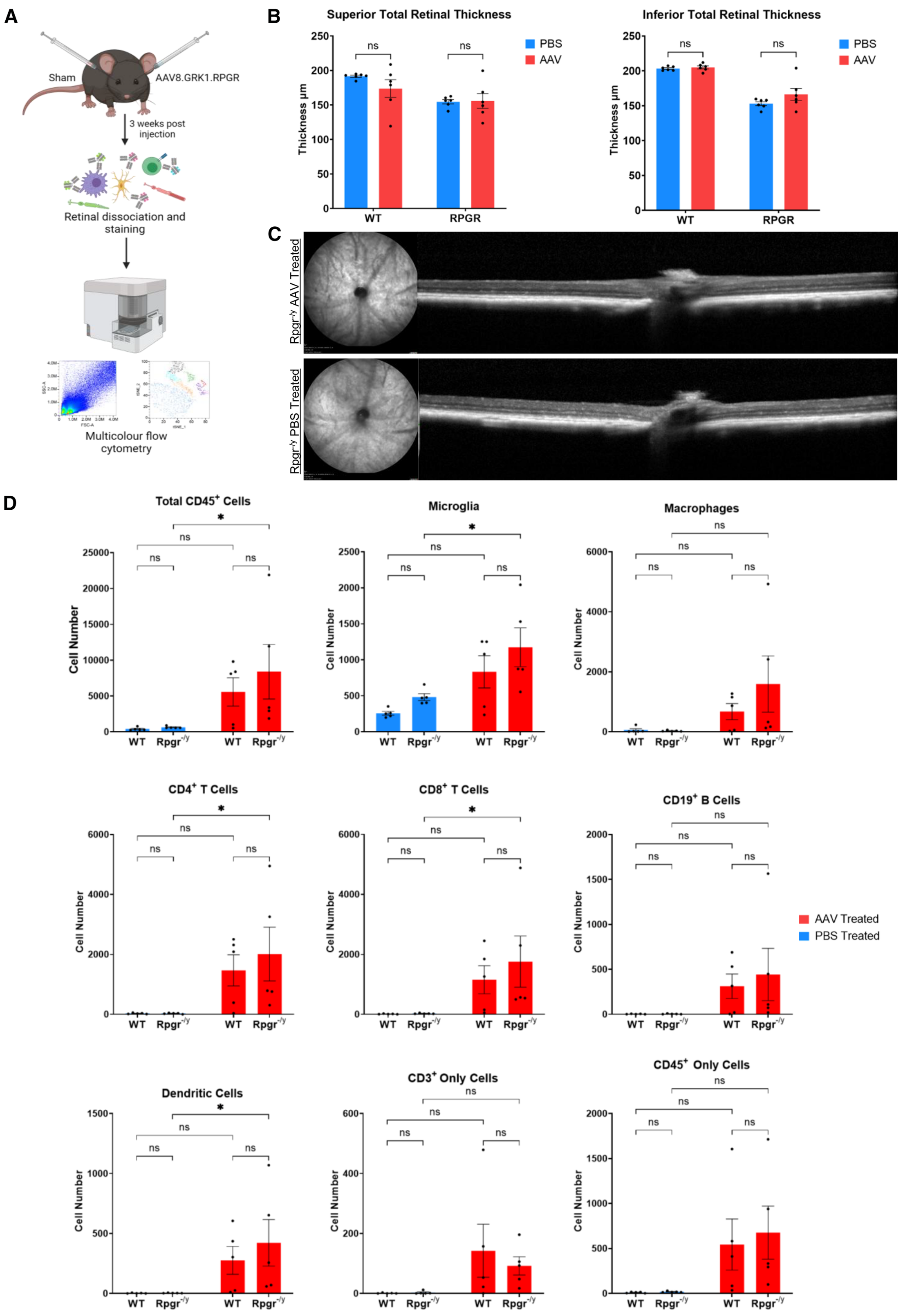

(legend on next page)

course of disease in Rpgr$^{-/y}$ may result in minimal inflammatory responses due to a level of desensitization to these motifs.[47] In comparison, the more rapid and high level of immunogenic DAMP signals in Rho^{P23H} would instigate a more robust immune response as seen in increased cellular infiltration into the retina.

When subjected to subretinal AAV gene therapy, both models generated a cellular immune response. The nature of the cellular immune response appears consistent across both IRD models and wild-type mice, irrespective of serotype and transgene. All AAV-treated retinas primarily demonstrated a type 1 cell-mediated anti-viral immune response, involving a large proportion of CD4 Th1 cells, consistent with previous studies in the retina. This response proved irrespective of genetic background or vector preparation.[28] Additionally, despite differences between the two vector preparations used in this study, no significant difference was seen in CD45^{+} cell numbers in wild-type mice that received paired injections of both vectors (Figure S7). In all treated eyes, CD4^{+} T cells represented the greatest immune cell infiltrate population after mononuclear phagocytes. These observations support the hypothesis that even subclinical inflammation post-gene therapy may adversely affect clinical outcomes, with Th1 responses geared toward viral capsids and potential destruction of transduced cells. In contrast to previous studies, we identified minor CD11c-positive dendritic cell and B cell populations in both AAV-treated IRD models and wild-type mice. In all groups, these represented small populations, suggesting that dendritic cells are unlikely to play a key antigen-presenting role in GTAU. Differences in retinal dissociation method compared to previous studies may account for better detection of these populations due to greater retention of cell surface markers (see materials and methods).[28] The uniformity in the nature of the immunological response to AAV across different models underscores the potential for developing universal immunomodulatory therapies that could mitigate GTAU and improve clinical outcomes of retinal gene therapy. By targeting key elements of the anti-viral immune pathway and controlling both clinical and subclinical retinal inflammation, the safety and efficacy of gene therapy may be improved. In this study, we did not assess the systemic adaptive immune response to subretinal AAV gene therapy. It would be valuable in future investigations to explore systemic immune biomarkers that might guide the mode and duration of immunosuppression in patients undergoing gene therapy.

Despite the difference in absolute magnitude of increase in retinal immune cells between *Rpgr*$^{-/y}$ or wild-type mice versus Rho^{P23H} mice, the relative fold change from baseline in each strain appeared comparable. This suggests that all three strains manifest similar nature of immune response to subretinal AAV. However, the greater background level of retinal inflammation in the Rho^{P23H} mice would mean that the post-gene therapy retinal immune cell populations could reach a higher magnitude, which becomes clinically manifest. Clinically, the post-gene therapy peak level of immune cell infiltration would determine if intraocular inflammation remains sub-clinical or clinically visible (e.g., in the form of visible subretinal infiltrates on OCT).

A range of clinical manifestations of GTAU have been observed in clinical trials and real-world deployment of AAV-mediated retinal gene therapy.[9] Our findings would support a more tailored approach to immunosuppression or immunomodulation depending on the rate of retinal degeneration and background immune status of the individual. For instance, relatively static conditions, such as achromatopsia, might be associated with milder background retinal inflammation than progressive IRDs such as LCA, RP, and choroideremia. Moreover, patients with more severe mutations within the same gene—such as null mutations versus missense mutations—may experience more rapid degeneration, thus carrying an increased risk for inflammation following gene therapy. Further investigation of immune responses in a wider range of IRDs would help to elucidate the corresponding risks of GTAU. Our findings would also support potential stratification of patients based on the background immune state of the retina when assessing suitability for gene therapy enrollment, for instance, ensuring that patients with a high risk of adverse immune responses are excluded from high-dose cohorts, which carry additional risk for GTAU. Patients with signs of ocular inflammation, such as vitreous cells, epiretinal membrane, or those demonstrating more rapid degeneration, may benefit from stronger or more prolonged immunosuppression to minimize their risk of adverse outcomes. Interestingly, our data also suggest a potential difference between male and female immune backgrounds in IRDs, as well as differences in immune response to AAV gene therapy, though the latter did not reach statistical significance with a small sample size (Figure S6). This supports differences in ocular inflammatory response between ages and sex recently reported by Clare et al., who observed greater divergence in microglial responses to AAV in females across age.[48] This observation warrants further investigation, and future clinical trials should consider better reporting of patient sex correlated to adverse inflammatory events to help understand its influence on treatment outcomes. Overall, our findings support more detailed patient stratification and reporting of relevant risk factors in retinal gene therapy trials to prevent GTAU and improve visual outcomes.

MATERIALS AND METHODS

Animals

All animal work have been approved by the institutional ethics review board and UK Home Office under project licence. Wild-type

Figure 7. Subretinal gene therapy induces similar levels of immune response in *Rpgr*$^{-/y}$ and wild-type mice

(A) Schematic workflow: subretinal injection of an AAV8.GRK1.hRPGR vector or sham (phosphate-buffered saline) in paired eyes, followed by *in vivo* SLO/OCT and flow cytometric analysis of dissociated retinas at 3 weeks post injection. (B) Changes in retinal thickness following subretinal treatment indicate no vector-related retinal toxicity. Two-way ANOVA with Šídák's correction (ns: not significant). (C) Post-treatment OCT of AAV vector and sham-injected retinas did not show any vitritis or significant retinal structural changes. (D) Comparison of immune cell populations before and after subretinal AAV gene therapy between *Rpgr*$^{-/y}$ (n = 5) and age-matched wild-type (n = 5) mice. Two-way repeated measures ANOVA uncorrected Fisher's LSD (ns: not significant).

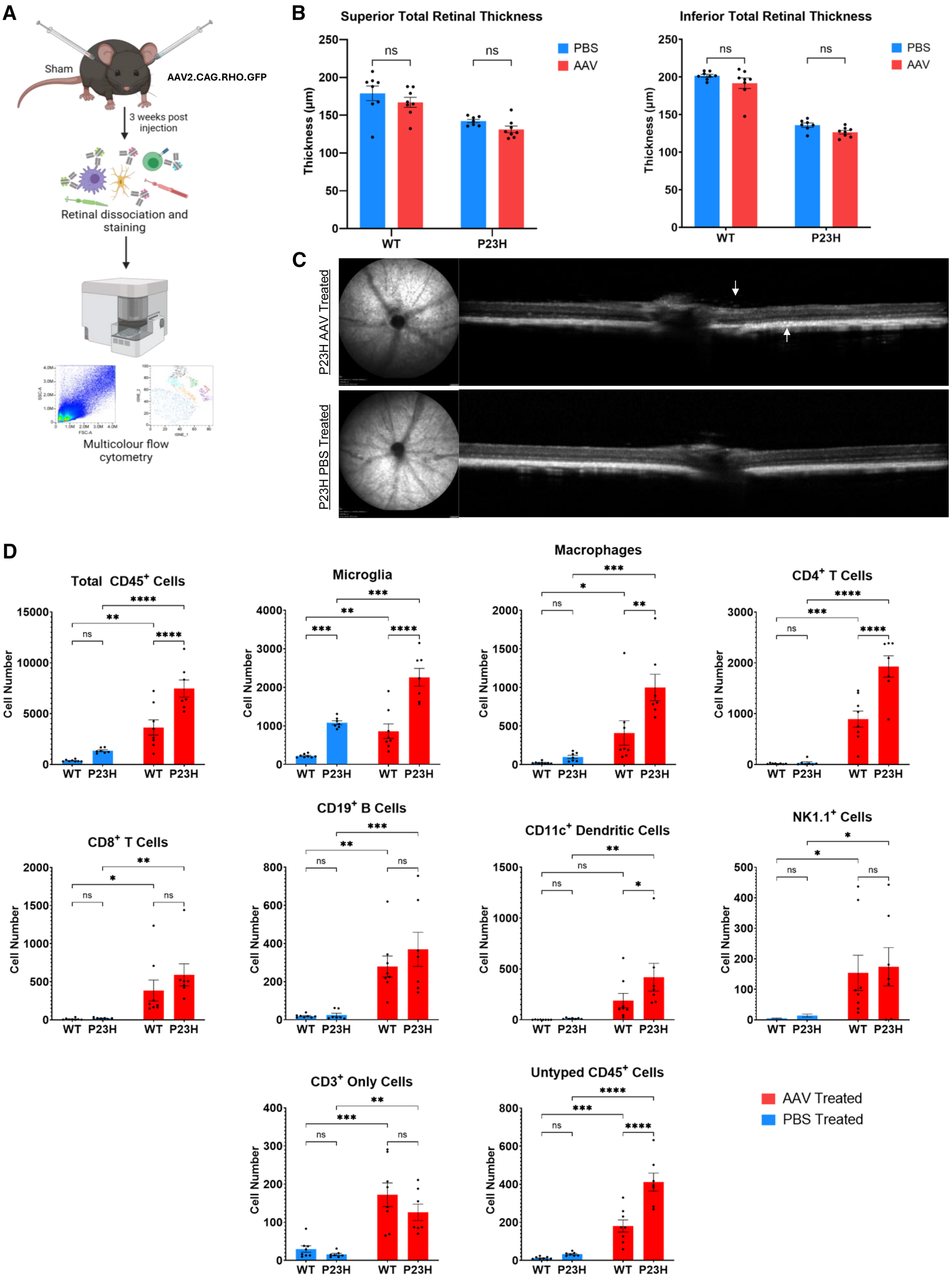

Figure 8. Subretinal gene therapy induces exacerbated immune cell infiltration in Rho(P23H) mice

(A) Schematic workflow for assessment of cellular immune response to subretinal injection of an AAV2.CAG.RHO.eGFP vector or sham (PBS) in paired eyes. (B) Treatment-induced changes in retinal thickness as determined by OCT did not demonstrate any vector-related retinal toxicity. (C) Post-treatment OCT of AAV vector and sham-injected

(legend continued on next page)

C57BL6/J (WT) mice were purchased from Charles River. Rho^{P23H} mice (B6.129S6(Cg)-*Rho*$^{tm1.1Kpal}$/J) were previously purchased from The Jackson Laboratory and a colony maintained at the University of Oxford Biomedical Sciences Division. Frozen embryos of *Rpgr*$^{-/y}$ mice were a kind gift previously obtained under material transfer agreement from Tiansen Li (Neurobiology, Neurodegeneration & Repair Laboratory, NEI, Bethesda, USA).

All animals were housed in the Biomedical Sciences Division, University of Oxford, in a 12 h light-dark cycle environment with food and water constantly available. All animal procedures were undertaken in accordance with the Association for Research in Vision and Ophthalmology guidelines for the humane use of laboratory animals in ophthalmic research and were approved by local and national ethical and legal authorities.

SLO and OCT assessment

Mice were anesthetized (ketamine [Narketan, Vetoquinol] [8 mg/mL] and xylazine [Rompun, Bayer] [1 mg/mL] in 0.9% saline at a volume of 0.1 mL per 10 g bodyweight) and pupils were dilated (phenylephrine hydrochloride 2.5% w/v and tropicamide 1% w/v) prior to imaging. Eyes were moistened with a drop of hypromellose 0.3% w/v (Blumont Healthcare Ltd.) and a contact lens of specification 3.2 mm diameter, 1.7 mm back optic zone radius, 0.4 mm central thickness, and plano refractive power (Cantor & Nissel Ltd.) was placed on the eye ensuring good visualization of the pupil.

Scanning laser ophthalmoscopy (SLO) and OCT images were acquired using the Spectralis ophthalmic imaging platform (Heidelberg Engineering) with a 55° lens. Retinal measurements were taken in the Spectralis software (Heidelberg) from SD-OCT scans in the outer retina at a set distance of 3 mm from the optic nerve head. Data are presented as an average superior (superior, superior temporal, and superior nasal) and inferior (inferior, inferior temporal, and inferior nasal) thickness per eye.

AAV production

AAV8.GRK1.hRPGR, research grade vector, was produced at Nationwide Children's Hospital, Columbus, Ohio, via transient transfection of HEK cells. The vector contains the human, codon-optimized RP GTPase regulator (RPGR) sequence under the control of the ubiquitous synthetic CAG promoter (hybrid of the CMV immediate enhancer and chicken beta-actin [β-actin] promoter).

AAV2.CAG.RHO.GFP was produced via transient transfection of adherent HEK293T cells. Confluent HYPERflasks (Corning) were transfected with the pDG RepCap plasmid (Plasmid Factory) of appropriate serotype and the transgene plasmid in TransIT-VirusGEN Transfection Reagent (Mirus). HYPERflasks were harvested 72 h post transfection, and the cell pellet was lysed (lysis buffer 1 M Tris, 150 mM NaCl, cOmplete Protease Inhibitor [Roche] in water, pH 8.5). Lysate was subjected to three freeze-thaw samples and benzonase (Merck) treated. AAV particles were isolated via an ultracentrifugation iodixanol gradient and concentrated and buffer exchanged for PBS with an Amicon Ultra 100k filter unit (Millipore). Titer was determined via qPCR with primers specific to the transgene (primers in supplemental). The vector contains the human rhodopsin (RHO) sequence and the GFP sequence under the control of the tissue-specific human rhodopsin kinase (GRK1) promoter.

Subretinal injection

Anesthetized and dilated mice received proxymetacaine hydrochloride 0.5% eye drops (Bausch & Lomb, UK) for local anesthesia. Paracentesis was performed via puncture of the peripheral cornea with a 33G needle to reduce ocular pressure and minimize the risk of reflux of the injection material. A large quantity of Viscotears Liquid Gel (0.2 mg/g polyacrylic acid) (Alcon) was applied to the cornea, and a 5 mm glass coverslip was placed over the eye to allow for visualization of the optic nerve head. The animal was positioned for viewing under a foot pedal-controlled Leica microsurgical operating microscope.

Injection material was loaded into a sterile NanoFil 10 μL syringe, which was attached to a 35G NanoFil needle (both World Precision 74 Instruments). For assessment in *Rpgr*$^{-/y}$ mice, AAV8.GRK1.hRPGR (1.5×10^9 vg in 1.5 μL) was injected in the right eye and 1.5 μL vehicle control (sterile PBS) in the left eye. For assessment of P23H mice, AAV2.CAG.RHO.GFP (1.5 x 10^9 in 1 μL) was injected in the right eye and 1 μL vehicle control (sterile PBS) in the left eye.

The eye was stabilized via grasping the superior rectus muscle with forceps, and the needle passed through the sclera into the subretinal space in the superior. The needle bevel was directly observed to be under the retina. Injection was performed to create a subretinal bleb with a bullous superior hemi-retinal detachment. Surgical complications (e.g., hemorrhage, extraocular reflux of material, and accidental intravitreal injection) were recorded at the time of injection, and affected animals were excluded from analysis.

Post injection, the needle was withdrawn, the coverslip was removed, and a drop of the antibiotic chloramphenicol 0.5% w/v (Minims, Bausch & Lomb) was applied. Viscotears was applied to hydrate the cornea during recovery.

Flow cytometry

Animals were sacrificed via a recognized schedule 1 method, and eyes were enucleated. The retina was removed via blunt dissection

retinas did not reveal any significant retinal structural changes. Mild vitritis and subretinal infiltrates were seen in AAV-injected eyes (indicated). (D) Comparison of immune cell populations before and after subretinal AAV gene therapy between Rho^{P23H} ($n = 7$) and age-matched wild-type ($n = 8$) mice. The Rho^{P23H} mice demonstrated significantly stronger increase in immune cell (CD45$^+$) infiltration post-gene therapy compared with wild-type controls. These mainly consisted of monocytic phagocytes (either macrophages or microglia) and CD4 T cells. Two-way repeated measures ANOVA uncorrected Fisher's LSD, $^{**}p < 0.01$, $^{***}p < 0.001$, $^{****}p < 0.0001$, ns: not significant.

and mechanically dissociated followed by resuspension in cell staining buffer (PBS, 2% FBS, 2 mM EDTA, 0.1% sodium azide). Samples were blocked with TruStain FcX Plus and Tru-Stain Monocyte Blocker prior to staining with primary fluorophore-conjugated antibodies (all BioLegend) (Table S2). Samples were incubated for 20 min, washed three times, and 7-AAD Viability Dye was added.

All flow cytometry samples were run on the Cytek Aurora 5 Laser (16UV-16V-14B-10YG-8R) with an event limit of 500,000 total events per retinal sample run. All data analysis was performed using the FlowJo software (v.10.10.0; BD Life Sciences). Gating for each experiment is outlined in supplemental figures.

IHC

Mice were euthanized, eyes enucleated, and cleaned of excess tissue. Whole eyes were fixed in 4% paraformaldehyde on ice for 20 min, followed by removal of the cornea and lens via dissection. Remaining posterior eyecups were cryoprotected in a sucrose gradient (10%, 20%, and 30%) at 4°C. Eyecups were placed in optimal cutting temperature medium and frozen in molds on dry ice.

16 μm sections were cut at −20°C with a cryotome and placed onto Superfrost plus slides (VWR). Slides were washed in PBS, and antigen retrieval was performed in citric acid buffer where appropriate. Slides were blocked (PBS, 10% BSA, 10% serum of the secondary antibody host, 0.1% Triton) and then incubated with primary antibodies (Table S3) overnight at 4°C in a solution containing 1% BSA and 1% serum in PBS. Slides were washed with 0.05% Tween 20 in PBS and rinsed in PBS. Appropriate secondary antibodies were stained under dark conditions for 2 h at room temperature. Slides were then briefly washed with 0.05% Tween 20 in PBS before counterstaining with Hoechst for 30 min in the dark. Coverslips were mounted with SlowFade Diamond Antifade Mountant (Thermo Fisher Scientific) and sealed. z stack images of sections were captured on a Fluoview FV3000 (Olympus) confocal microscope and analyzed in ImageJ (Fiji). Qualitative assessments of the IHC sections were determined by three independent investigators who agreed to each conclusion.

For TUNEL staining, the ApopTag Red In-Situ Apoptosis Detection Kit (Sigma-Aldrich) was used as per the manufacturer's instructions.

RPE flat mounts

Mice were euthanized, eyes enucleated, and cleaned of excess tissue. Whole eyes were fixed in 4% paraformaldehyde on ice for 10 min, followed by removal of the cornea and lens via dissection. The retina was then carefully removed. Remaining posterior eyecups were submerged in blocking buffer PBS, 10% BSA, and 10% serum of the secondary antibody host (0.1% Triton) and then incubated with primary antibodies (Table S3) overnight at 4°C in a solution containing 1% BSA and 1% serum in PBS. Eyecups were washed with 0.05% Tween 20 in PBS and rinsed in PBS. Appropriate secondary antibodies were stained under dark conditions for 2 h at room temperature. Tissue was briefly washed with 0.05% Tween 20 in PBS before counterstaining with Hoechst for 30 min in the dark. Eyecups were then placed on slides, and radial cuts were made to allow tissue to lay flat. Coverslips were mounted with SlowFade Diamond Antifade Mountant (Thermo Fisher Scientific) and sealed. z stack images of sections were captured on a Fluoview FV3000 (Olympus) confocal microscope and analyzed in ImageJ (Fiji). ZO-1 staining was assessed in ImageJ as area of positive stain above background threshold across acquired images (n = 4 eyes, 4 fields per eye).

Data analysis

Data were presented and analyzed in GraphPad Prism v.10.3.1 with Two-way repeated measures ANOVA uncorrected Fisher's least significant difference (LSD) or two-tailed unpaired t tests as stated in figure legends. Graphs indicate the mean ± SEM unless otherwise stated.

DATA AND CODE AVAILABILITY

Raw data can be made available upon request.

ACKNOWLEDGMENTS

The authors would like to acknowledge funding from the Biotechnology and Biological Sciences Research Council (BBSRC) Collaborative Training Programme (CTP) in Advanced Bioscience of Viral Products (ABViP) (M.C.J.; BB/X511353/1), Wellcome Trust (K.X.; 216593/Z/19/Z), UKRI Medical Research Council (MRC) (H.S., J.C.-K., and A.D.D.), and National Institute for Health Research (NIHR) Oxford Biomedical Research Centre (BRC) (C.M.-F.d.l.C., A.S., M.E.M., M.D.F., and R.E.M.). A.B. is an employee of Oxford Biomedica Ltd. The graphical abstract and elements of Figures 6 and 7 were made with Biorender.

AUTHOR CONTRIBUTIONS

The study was designed by M.C.J. and K.X. Research was performed by M.C.J., C.M.-F.d.l.C., F.S., A.S., and H.S. The data were analyzed by M.C.J., C.M.-F.d.l.C., and K.X. The main manuscript text was written by M.C.J. and K.X. with contributions from C.M.-F.d.l.C., A.S., H.S., M.E.M., J.C.-K., A.B., M.D.F., A.D.D., and R.E.M. Figures were prepared by M.C.J., C.M.-F.d.l.C., and K.X.

DECLARATION OF INTERESTS

The authors declare no competing interests.

SUPPLEMENTAL INFORMATION

Supplemental information can be found online at https://doi.org/10.1016/j.omta.2025.201652.

REFERENCES

1. Fischer, M.D., Simonelli, F., Sahni, J., Holz, F.G., Maier, R., Fasser, C., Suhner, A., Stiehl, D.P., Chen, B., Audo, I., et al. (2024). Real-World Safety and Effectiveness of Voretigene Neparvovec: Results up to 2 Years from the Prospective, Registry-Based PERCEIVE Study. Biomolecules *14*, 122. https://doi.org/10.3390/BIOM14010122/S1.

2. Russell, S., Bennett, J., Wellman, J.A., Chung, D.C., Yu, Z.F., Tillman, A., Wittes, J., Pappas, J., Elci, O., McCague, S., et al. (2017). Efficacy and safety of voretigene neparvovec (AAV2-hRPE65v2) in patients with RPE65-mediated inherited retinal dystrophy: a randomised, controlled, open-label, phase 3 trial. Lancet *390*, 849–860. https://doi.org/10.1016/S0140-6736(17)31868-8.

3. Simonelli, F., Maguire, A.M., Testa, F., Pierce, E.A., Mingozzi, F., Bennicelli, J.L., Rossi, S., Marshall, K., Banfi, S., Surace, E.M., et al. (2010). Gene therapy for Leber's congenital amaurosis is safe and effective through 1.5 years after vector administration. Mol. Ther. *18*, 643–650. https://doi.org/10.1038/MT.2009.277.

4. Maguire, A.M., Simonelli, F., Pierce, E.A., Pugh, E.N., Mingozzi, F., Bennicelli, J., Banfi, S., Marshall, K.A., Testa, F., Surace, E.M., et al. (2008). Safety and efficacy

of gene transfer for Leber's congenital amaurosis. N. Engl. J. Med. *358*, 2240–2248. https://doi.org/10.1056/NEJMOA0802315.

5. Xue, K., Jolly, J.K., Barnard, A.R., Rudenko, A., Salvetti, A.P., Patrício, M.I., Edwards, T.L., Groppe, M., Orlans, H.O., Tolmachova, T., et al. (2018). Beneficial effects on vision in patients undergoing retinal gene therapy for choroideremia. Nat. Med. *24*, 1507–1512. https://doi.org/10.1038/s41591-018-0185-5.

6. Cehajic-Kapetanovic, J., Xue, K., Martinez-Fernandez de la Camara, C., Nanda, A., Davies, A., Wood, L.J., Salvetti, A.P., Fischer, M.D., Aylward, J.W., Barnard, A.R., et al. (2020). Initial results from a first-in-human gene therapy trial on X-linked retinitis pigmentosa caused by mutations in RPGR. Nat. Med. *26*, 354–359. https://doi.org/10.1038/s41591-020-0763-1.

7. Michaelides, M., Laich, Y., Wong, S.C., Oluonye, N., Zaman, S., Kumaran, N., Kalitzeos, A., Petrushkin, H., Georgiou, M., Tailor, V., et al. (2025). Gene therapy in children with AIPL1-associated severe retinal dystrophy: an open-label, first-in-human interventional study. Lancet *405*, 648–657. https://doi.org/10.1016/S0140-6736(24)02812-5.

8. McClements, M.E., Elsayed, M.E.A.A., Major, L., de la Camara, C.M.F., and MacLaren, R.E. (2024). Gene Therapies in Clinical Development to Treat Retinal Disorders. Mol. Diagn. Ther. *28*, 575–591. https://doi.org/10.1007/S40291-024-00722-0/TABLES/2.

9. Purdy, R., John, M., Bray, A., Clare, A.J., Copland, D.A., Chan, Y.K., Henderson, R.H., Nerinckx, F., Leroy, B.P., Yang, P., et al. (2025). Gene Therapy-Associated Uveitis (GTAU): Understanding and mitigating the adverse immune response in retinal gene therapy. Prog. Retin. Eye Res. *106*, 101354. https://doi.org/10.1016/J.PRETEYERES.2025.101354.

10. Bainbridge, J.W.B., Mehat, M.S., Sundaram, V., Robbie, S.J., Barker, S.E., Ripamonti, C., Georgiadis, A., Mowat, F.M., Beattie, S.G., Gardner, P.J., et al. (2015). Long-Term Effect of Gene Therapy on Leber's Congenital Amaurosis. N. Engl. J. Med. *372*, 1887–1897. https://doi.org/10.1056/NEJMOA1414221/SUPPL_FILE/NEJMOA1414221_DISCLOSURES.PDF.

11. Reichel, F.F., Dauletbekov, D.L., Klein, R., Peters, T., Ochakovski, G.A., Seitz, I.P., Wilhelm, B., Ueffing, M., Biel, M., Wissinger, B., et al. (2017). AAV8 Can Induce Innate and Adaptive Immune Response in the Primate Eye. Mol. Ther. *25*, 2648–2660. https://doi.org/10.1016/j.ymthe.2017.08.018.

12. Reichel, F.F., Seitz, I., Wozar, F., Dimopoulos, S., Jung, R., Kempf, M., Kohl, S., Kortüm, F.C., Ott, S., Pohl, L., et al. (2023). Development of retinal atrophy after subretinal gene therapy with voretigene neparvovec. Br. J. Ophthalmol. *107*, 1331–1335. https://doi.org/10.1136/BJOPHTHALMOL-2021-321023.

13. Seitz, I.P., Wozar, F., Ochakovski, G.A., Reichel, F.F., Gelisken, F., Bartz-Schmidt, K.U., Peters, T., and Fischer, M.D. (2024). Dose-Dependent Progression of Chorioretinal Atrophy at the Injection Site After Subretinal Injection of rAAV2/8 in Nonhuman Primates. Ophthalmol. Sci. *4*, 100516. https://doi.org/10.1016/J.XOPS.2024.100516.

14. Olivares-González, L., Velasco, S., Campillo, I., and Rodrigo, R. (2021). Retinal Inflammation, Cell Death and Inherited Retinal Dystrophies. Int. J. Mol. Sci. *22*, 2096. https://doi.org/10.3390/IJMS22042096.

15. Massengill, M.T., Ahmed, C.M., Lewin, A.S., and Ildefonso, C.J. (2018). Neuroinflammation in Retinitis Pigmentosa, Diabetic Retinopathy, and Age-Related Macular Degeneration: A Minireview. Adv. Exp. Med. Biol. *1074*, 185–191. https://doi.org/10.1007/978-3-319-75402-4_23.

16. Sarici, K., Vyas, A., and Iannaccone, A. (2023). The double-edged sword of inflammation in inherited retinal degenerations: Clinical and preclinical evidence for mechanistically and prognostically impactful but treatable complications. Front. Cell Dev. Biol. *11*, 1177711. https://doi.org/10.3389/FCELL.2023.1177711/FULL.

17. Adamus, G. (2021). Importance of Autoimmune Responses in Progression of Retinal Degeneration Initiated by Gene Mutations. Front. Med. *8*, 672444. https://doi.org/10.3389/FMED.2021.672444.

18. Okita, A., Murakami, Y., Shimokawa, S., Funatsu, J., Fujiwara, K., Nakatake, S., Koyanagi, Y., Akiyama, M., Takeda, A., Hisatomi, T., and Ikeda, Y. (2020). Changes of serum inflammatory molecules and their relationships with visual function in retinitis pigmentosa. Investig. Ophthalmol. Vis. Sci. *61*, 30. https://doi.org/10.1167/iovs.61.11.30.

19. Ten Berge, J.C., Fazil, Z., van den Born, I., Wolfs, R.C.W., Schreurs, M.W.J., Dik, W.A., and Rothova, A. (2019). Intraocular cytokine profile and autoimmune reactions in retinitis pigmentosa, age-related macular degeneration, glaucoma and cataract. Acta Ophthalmol. *97*, 185–192. https://doi.org/10.1111/aos.13899.

20. Yoshida, N., Ikeda, Y., Notomi, S., Ishikawa, K., Murakami, Y., Hisatomi, T., Enaida, H., and Ishibashi, T. (2013). Clinical evidence of sustained chronic inflammatory reaction in retinitis pigmentosa. Ophthalmology *120*, 100–105. https://doi.org/10.1016/J.OPHTHA.2012.07.006.

21. Appelbaum, T., Santana, E., and Aguirre, G.D. (2017). Strong upregulation of inflammatory genes accompanies photoreceptor demise in canine models of retinal degeneration. PLoS One *12*, e0177224. https://doi.org/10.1371/JOURNAL.PONE.0177224.

22. Chen, M., Luo, C., Zhao, J., Devarajan, G., and Xu, H. (2019). Immune regulation in the aging retina. Prog. Retin. Eye Res. *69*, 159–172. https://doi.org/10.1016/J.PRETEYERES.2018.10.003.

23. Caspi, R.R. (2006). Ocular autoimmunity: The price of privilege? Immunol. Rev. *213*, 23–35. https://doi.org/10.1111/J.1600-065X.2006.00439.X.

24. Cheng, S.Y., and Punzo, C. (2022). Update on Viral Gene Therapy Clinical Trials for Retinal Diseases. Hum. Gene Ther. *33*, 865–878. https://doi.org/10.1089/HUM.2022.159/SUPPL_FILE/SUPP_TABLES1.XLSX.

25. Busbee, B., Boyer, D.S., Khanani, A.M., Wykoff, C.C., Pieramici, D.J., Regillo, C., Danzig, C.J., Joondeph, B.C., Major, J., Hoang, C., et al. (2021). Phase 1 Study of Intravitreal Gene Therapy with ADVM-022 for neovascular AMD (OPTIC Trial). Investig. Ophthalmol. Vis. Sci. *62*, 352.

26. Zhang, J., Zhang, J., Zhang, C., Zhang, J., Gu, L., Luo, D., and Qiu, Q. (2022). Diabetic Macular Edema: Current Understanding, Molecular Mechanisms and Therapeutic Implications. Cells *11*, 3362. https://doi.org/10.3390/CELLS11213362.

27. Buckley, T., MacLaren, R.E., and Kapetanovic, J.C. (2024). Meta-analysis of Gene Therapy Associated Uveitis (GTAU). Investig. Ophthalmol. Vis. Sci. *65*, 5312.

28. Chandler, L.C., McClements, M.E., Yusuf, I.H., Martinez-Fernandez de la Camara, C., MacLaren, R.E., and Xue, K. (2021). Characterizing the cellular immune response to subretinal AAV gene therapy in the murine retina. Mol. Ther. Methods Clin. Dev. *22*, 52–65. https://doi.org/10.1016/j.omtm.2021.05.011.

29. Hong, D.-H., Pawlyk, B.S., Shang, J., Sandberg, M.A., Berson, E.L., and Li, T. (2000). A retinitis pigmentosa GTPase regulator (RPGR)- deficient mouse model for X-linked retinitis pigmentosa (RP3). Proc. Natl. Acad. Sci. USA *97*, 3649–3654. https://doi.org/10.1073/PNAS.060037497.

30. Cehajic-Kapetanovic, J., Martinez-Fernandez de la Camara, C., Birtel, J., Rehman, S., McClements, M.E., Charbel Issa, P., Lotery, A.J., and MacLaren, R.E. (2022). Impaired glutamylation of RPGRORF15 underlies the cone-dominated phenotype associated with truncating distal ORF15 variants. Proc. Natl. Acad. Sci. USA *119*, e2208707119. https://doi.org/10.1073/PNAS.2208707119/SUPPL_FILE/PNAS.2208707119.SAPP.PDF.

31. Mihailovic, N., Schimpf-Linzenbold, S., Sattler, I., Eter, N., and Heiduschka, P. (2022). The first reported case of a deletion of the entire RPGR gene in a family with X-linked retinitis pigmentosa. Ophthalmic Genet. *43*, 679–684. https://doi.org/10.1080/13816810.2022.2083181.

32. Di Iorio, V., Karali, M., Melillo, P., Testa, F., Brunetti-Pierri, R., Musacchia, F., Condroyer, C., Neidhardt, J., Audo, I., Zeitz, C., et al. (2020). Spectrum of Disease Severity in Patients With X-Linked Retinitis Pigmentosa Due to RPGR Mutations. Investig. Ophthalmol. Vis. Sci. *61*, 36. https://doi.org/10.1167/IOVS.61.14.36.

33. Díaz-Lezama, N., Kajtna, J., Wu, J., Ayten, M., and Koch, S.F. (2023). Microglial and macroglial dynamics in a model of retinitis pigmentosa. Vision Res *210*, 108268. https://doi.org/10.1016/J.VISRES.2023.108268.

34. Bringmann, A., Iandiev, I., Pannicke, T., Wurm, A., Hollborn, M., Wiedemann, P., Osborne, N.N., and Reichenbach, A. (2009). Cellular signaling and factors involved in Müller cell gliosis: neuroprotective and detrimental effects. Prog. Retin. Eye Res. *28*, 423–451. https://doi.org/10.1016/J.PRETEYERES.2009.07.001.

35. Vijayasarathy, C., Zeng, Y., Brooks, M.J., Fariss, R.N., and Sieving, P.A. (2021). Genetic Rescue of X-Linked Retinoschisis Mouse (Rs1−/y) Retina Induces Quiescence of the Retinal Microglial Inflammatory State Following AAV8-RS1 Gene Transfer and Identifies Gene Networks Underlying Retinal Recovery. Hum.

Gene Ther. *32*, 667–681. https://doi.org/10.1089/HUM.2020.213. https://home.liebertpub.com/hum.

36. Lam, B.L., Pennesi, M.E., Kay, C.N., Panda, S., Gow, J.A., Zhao, G., and MacLaren, R.E.; XIRIUS Study Group (2024). Assessment of Visual Function with Cotoretigene Toliparvovec in X-Linked Retinitis Pigmentosa in the Randomized XIRIUS Phase 2/3 Study. Ophthalmology *131*, 1083–1093. https://doi.org/10.1016/j.ophtha.2024.02.023.

37. Fischer, M.D., McClements, M.E., Martinez-Fernandez de la Camara, C., Bellingrath, J.S., Dauletbekov, D., Ramsden, S.C., Hickey, D.G., Barnard, A.R., and MacLaren, R.E. (2017). Codon-Optimized RPGR Improves Stability and Efficacy of AAV8 Gene Therapy in Two Mouse Models of X-Linked Retinitis Pigmentosa. Mol. Ther. *25*, 1854–1865. https://doi.org/10.1016/J.YMTHE.2017.05.005.

38. Langmann, T. (2007). Microglia activation in retinal degeneration. J. Leukoc. Biol. *81*, 1345–1351. https://doi.org/10.1189/JLB.0207114.

39. Rathnasamy, G., Foulds, W.S., Ling, E.A., and Kaur, C. (2019). Retinal microglia – A key player in healthy and diseased retina. Prog. Neurobiol. *173*, 18–40. https://doi.org/10.1016/J.PNEUROBIO.2018.05.006.

40. Jurga, A.M., Paleczna, M., and Kuter, K.Z. (2020). Overview of General and Discriminating Markers of Differential Microglia Phenotypes. Front. Cell. Neurosci. *14*, 198. https://doi.org/10.3389/FNCEL.2020.00198/PDF.

41. O'Koren, E.G., Mathew, R., and Saban, D.R. (2016). Fate mapping reveals that microglia and recruited monocyte-derived macrophages are definitively distinguishable by phenotype in the retina. Sci. Rep. *6*, 20636. https://doi.org/10.1038/srep20636.

42. O'Koren, E.G., Yu, C., Klingeborn, M., Wong, A.Y.W., Prigge, C.L., Mathew, R., Kalnitsky, J., Msallam, R.A., Silvin, A., Kay, J.N., et al. (2019). Microglial Function Is Distinct in Different Anatomical Locations during Retinal Homeostasis and Degeneration. Immunity *50*, 723–737.e7. https://doi.org/10.1016/J.IMMUNI.2019.02.007/ATTACHMENT/1B2C5836-80E9-40BE-80BB-A991A2ED2D8C/MMC2.PDF.

43. Hippert, C., Graca, A.B., Barber, A.C., West, E.L., Smith, A.J., Ali, R.R., and Pearson, R.A. (2015). Müller Glia Activation in Response to Inherited Retinal Degeneration Is Highly Varied and Disease-Specific. PLoS One *10*, e0120415. https://doi.org/10.1371/JOURNAL.PONE.0120415.

44. Graca, A.B., Hippert, C., and Pearson, R.A. (2018). Müller Glia Reactivity and Development of Gliosis in Response to Pathological Conditions. Adv. Exp. Med. Biol. *1074*, 303–308. https://doi.org/10.1007/978-3-319-75402-4_37.

45. Mahaling, B., Low, S.W.Y., Beck, M., Kumar, D., Ahmed, S., Connor, T.B., Ahmad, B., and Chaurasia, S.S. (2022). Damage-Associated Molecular Patterns (DAMPs) in Retinal Disorders. Int. J. Mol. Sci. *23*, 2591. https://doi.org/10.3390/IJMS23052591.

46. Karlen, S.J., Miller, E.B., and Burns, M.E. (2020). Microglia Activation and Inflammation during the Death of Mammalian Photoreceptors. Annu. Rev. Vis. Sci. *6*, 149–169. https://doi.org/10.1146/ANNUREV-VISION-121219-081730/CITE/REFWORKS.

47. Pradeu, T., Jaeger, S., and Vivier, E. (2013). The speed of change: towards a discontinuity theory of immunity? Nat. Rev. Immunol. *13*, 764–769. https://doi.org/10.1038/nri3521.

48. Clare, A.J., Langer, P.M., Ward, A., Chan, Y.K., Dick, A.D., and Copland, D.A. (2025). Characterization of the ocular inflammatory response to AAV reveals divergence by sex and age. Mol. Ther. *33*, 1246–1263. https://doi.org/10.1016/j.ymthe.2025.01.028.

OMTA, Volume *34*

Supplemental information

Pre-existing retinal inflammation exacerbates gene therapy-associated uveitis in more rapidly progressive retinal degeneration

Molly C. John, Cristina Martinez-Fernandez de la Camara, Ahmed Salman, Federica Staurenghi, Hoda Shamsnajafabadi, Michelle E. McClements, Jasmina Cehajic-Kapetanovic, Alissa Bray, M Dominik Fischer, Andrew D. Dick, Robert E. MacLaren, and Kanmin Xue

SUPPLEMENTAL MATERIAL

Table S1 -Primers for AAV Titration qPCR

Primer Name	Sequence	Target
GFPqPCR_F	ACTACCCTGACCTATGGCGT	GFP Transgene
GFPqPCR_R	CTCTCCTGCACATAGCCCT	GFP Transgene

Table S2 - List of markers and fluorophores used flow cytometric analysis of retinal immune cell populations

Target Cell Type	Antibody	Fluorophore	Staining Concentration (ug/ml)
All Leukocytes	CD45 30-F11	Brilliant Violet 711	0.5
Monocytes, Macrophage, dendritic cells, NK cell, granulocytes, B and T cell subsets	CD11b M1/70	APC	0.25
T cells, NK T cells	CD3 17A2	AlexaFluor 700	5
T cell subset	CD4 RM4-5	Brilliant Violet 785	0.5
B cells	CD19 6D5	APC/Fire 750	0.5
Natural Killer Cells	NK-1.1 PK136	PE/Cyanine7	1
Dendritic Cells	CD11c N418	PerCP/Cyanine5.5	1
T Cell subset	CD8a 53-6.7	PE	0.125

***Table S3* - Antibodies used for immunohistochemistry analysis of the retina.**

Antibody	Target	Concentration
Rabbit anti-IBA1 (Wako, WDE1198, 019-19741)	IBA1 - Microglia, Murine	1:500
Rabbit anti-GFAP (ab7260, Abcam)	GFAP - Muller Glia, Murine	1:500
Goat anti-GFAP (ab5353, Abcam)	GFAP - Muller Glia, Murine	1:500
Rat anti-CD45 (30-F11, Biolegend)	CD45 - All immune cells, Murine	1:200
Mouse anti-ZO1 (ZO1-1A12, Invitrogen)	ZO-1 – Tight junctions	1:200
Goat anti-CD68 (sc-7084, Santa Cruz Biotechnology)	CD68 - Microglia, Murine	1:100
Donkey anti-Mouse-AF647 (Biolegend)	Mouse IgG	1:400
Donkey anti-Rabbit-AF568 (Biolegend)	Rabbit IgG	1:400
Goat anti-Rat-AF568 (Biolegend)	Rat IgG	1:400
Donkey ant-Goat-AF647 (Biolegend)	Goat IgG	1:400

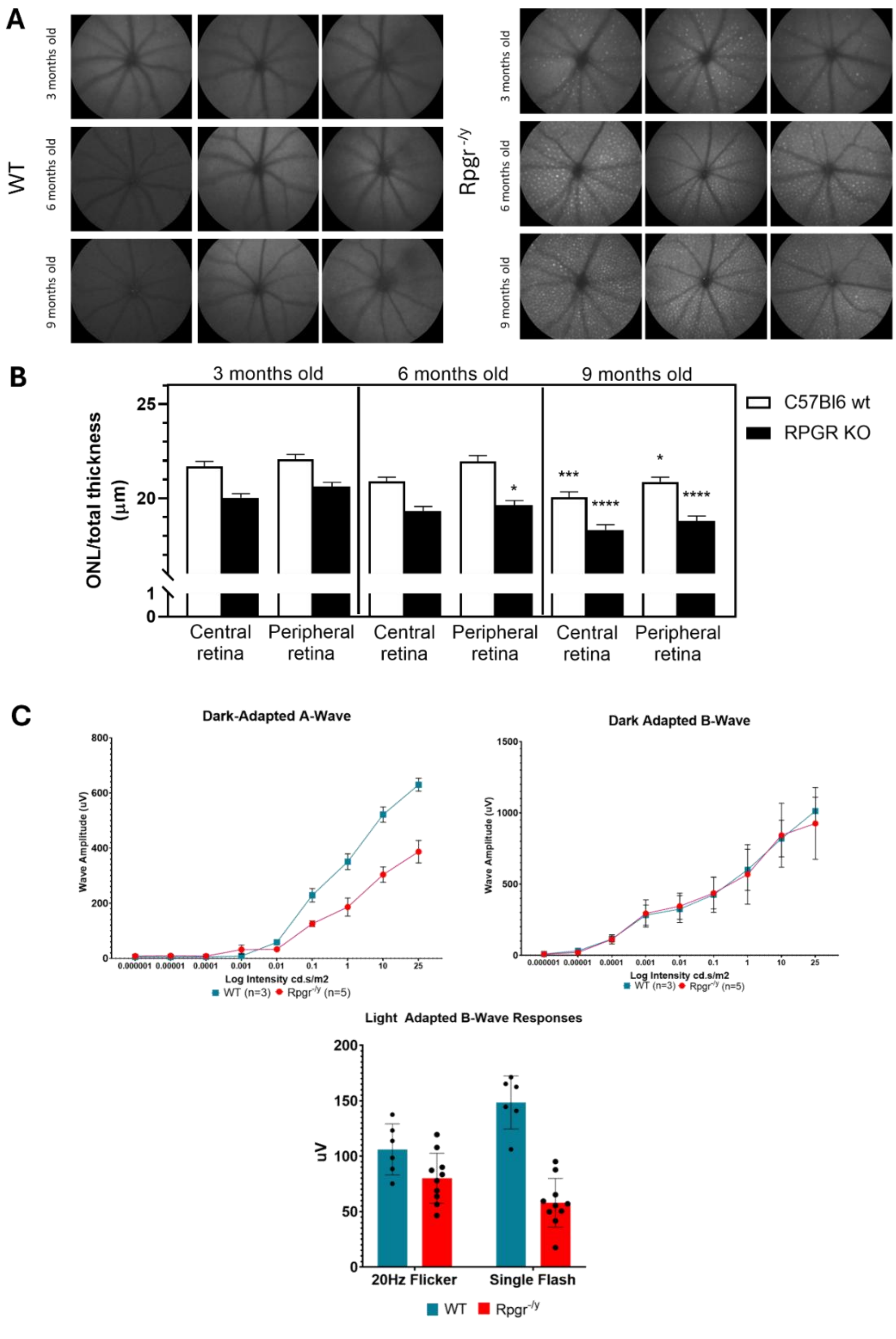

Figure S1. Rpgr$^{-/y}$ progressive phenotype 3-9 months. *(**A**) Progressive development of hyperreflective spots as seen on cSLO in Rpgr$^{-/y}$ versus C57BL6j WT mice from 3 to 9 months of age. (**B**) Progressive thinning of Rpgr$^{-/y}$ retinae from 3-9 months of age. Significance versus the same strain at 3 months of age determined by ANOVA (* p<0.05, *** p<0.001, **** p<0.0001). (**C**) Electroretinography assessment of 12-month old Rpgr$^{-/y}$ mice (n=5) versus age-matched controls (n=3).*

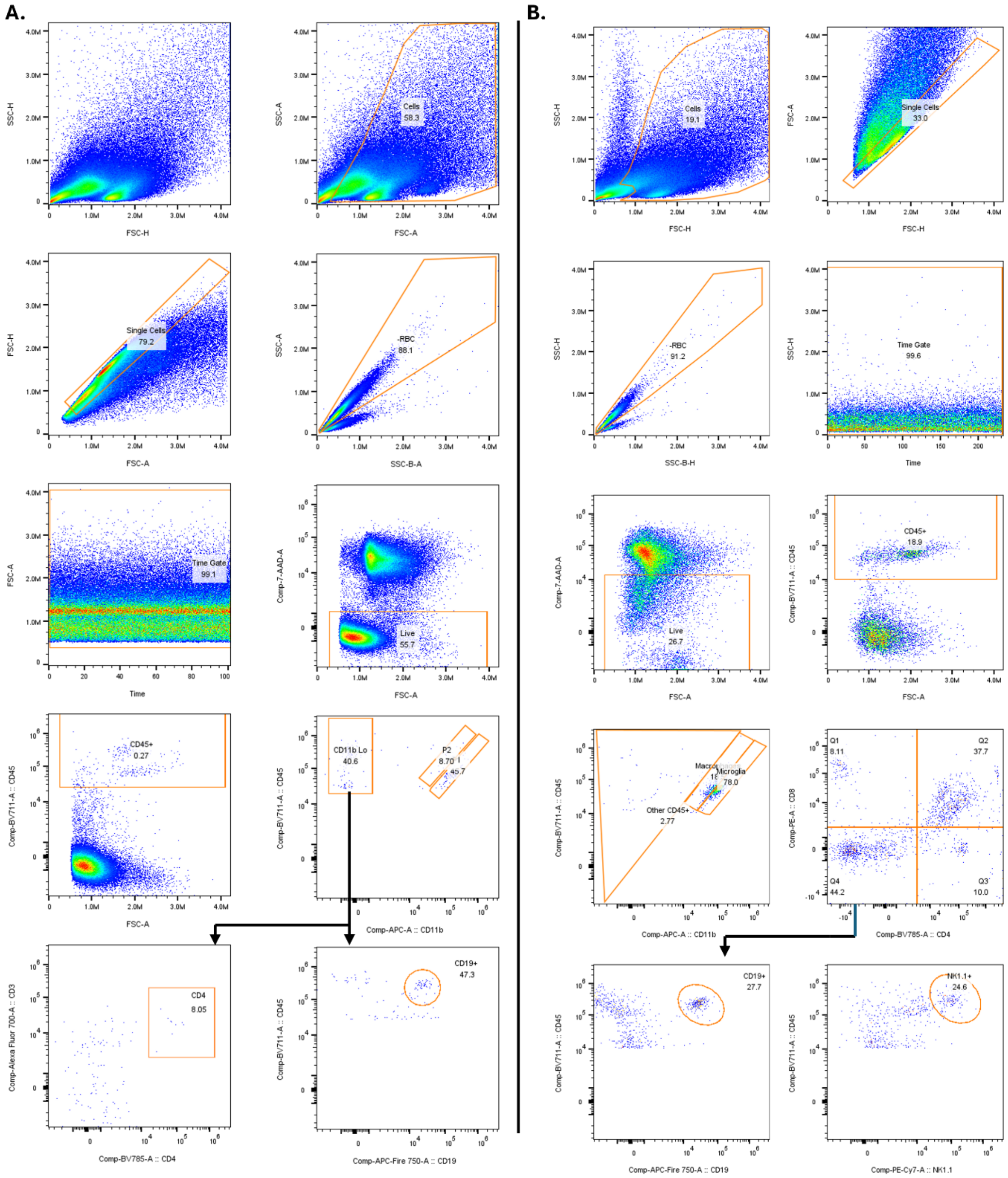

Figure S2. Rpgr$^{-/y}$ and Rho^{P23H} Immunotyping Gating Strategy. *Gating was performed subjectively on multicolour stained retinal samples from Rpgr$^{-/y}$, Rho^{P23H} and WT animals. (**A**) Rpgr$^{-/y}$ and corresponding WT gating. The same gates were applied across all samples, the gated population was brought forward into the next consecutive gate with the exception of the CD4 and CD19 gating which was performed on the population in the 'CD11b Lo' gate as indicated. (**B**) Rho^{P23H} and corresponding WT gating. The same gates were applied across all samples, the gated population was brought forward into the next consecutive gate unless stated. CD19 gating was performed on the population in the 'CD4-CD8-' Q4 gate as indicated. NK1.1 gating taken from a 'NOT' gate of CD19. To allow gating of rare events, gates were decided using a concated sample of all CD45 events in all samples. Gate statistics shown represent the proportion of plotted events within each gate. Gating analysis was performed in FlowJo v10.10.0*

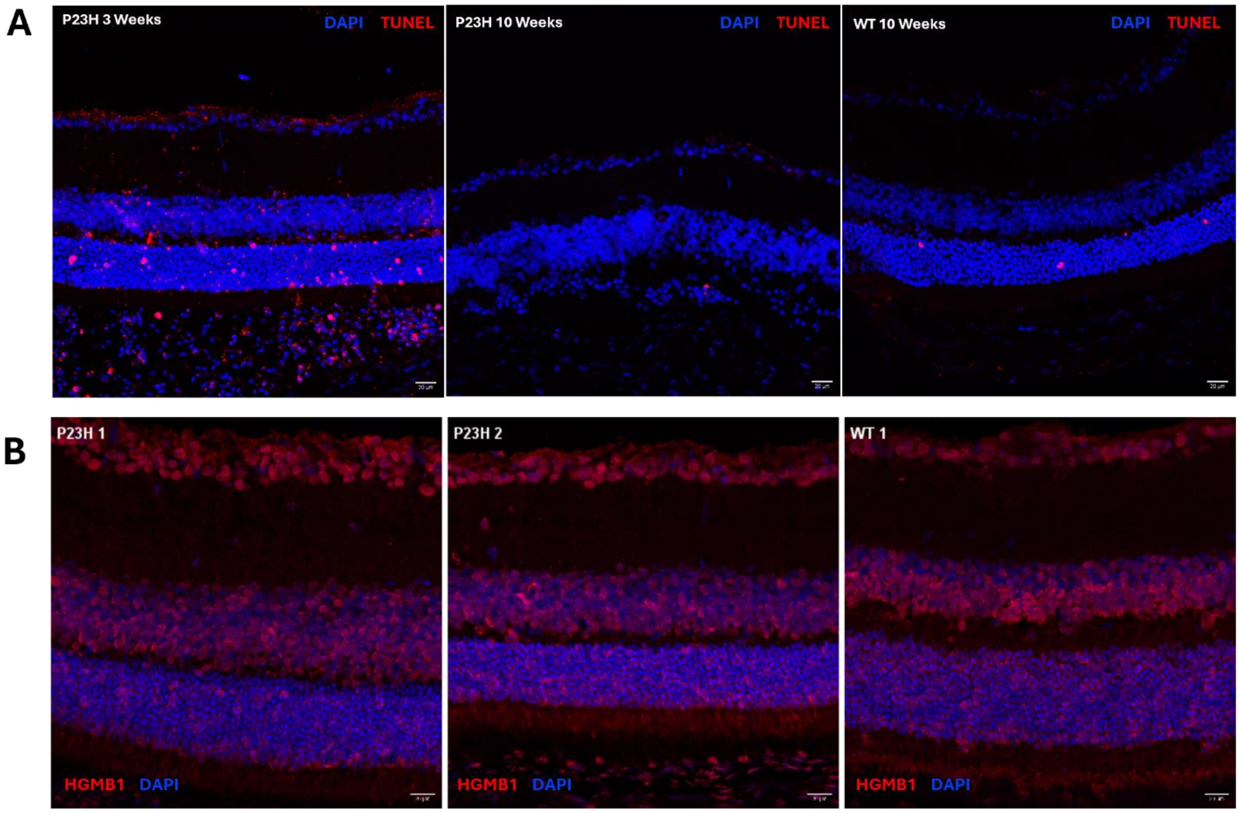

Figure S3. Apoptosis in Rho^{P23H} 3 and 10 week old mice and Rho^{P23H} HGMB1 Staining. (**A**) TUNEL staining in two representative Rho^{P23H} retinas at 3-weeks versus 10-weeks old shows extensive outer retina TUNEL positivity in the younger mice that decreases in older animals, combined with a loss of the ONL. (**B**) HGMB1 staining in two representative Rho^{P23H} retinas (3-weeks) versus the wild-type.

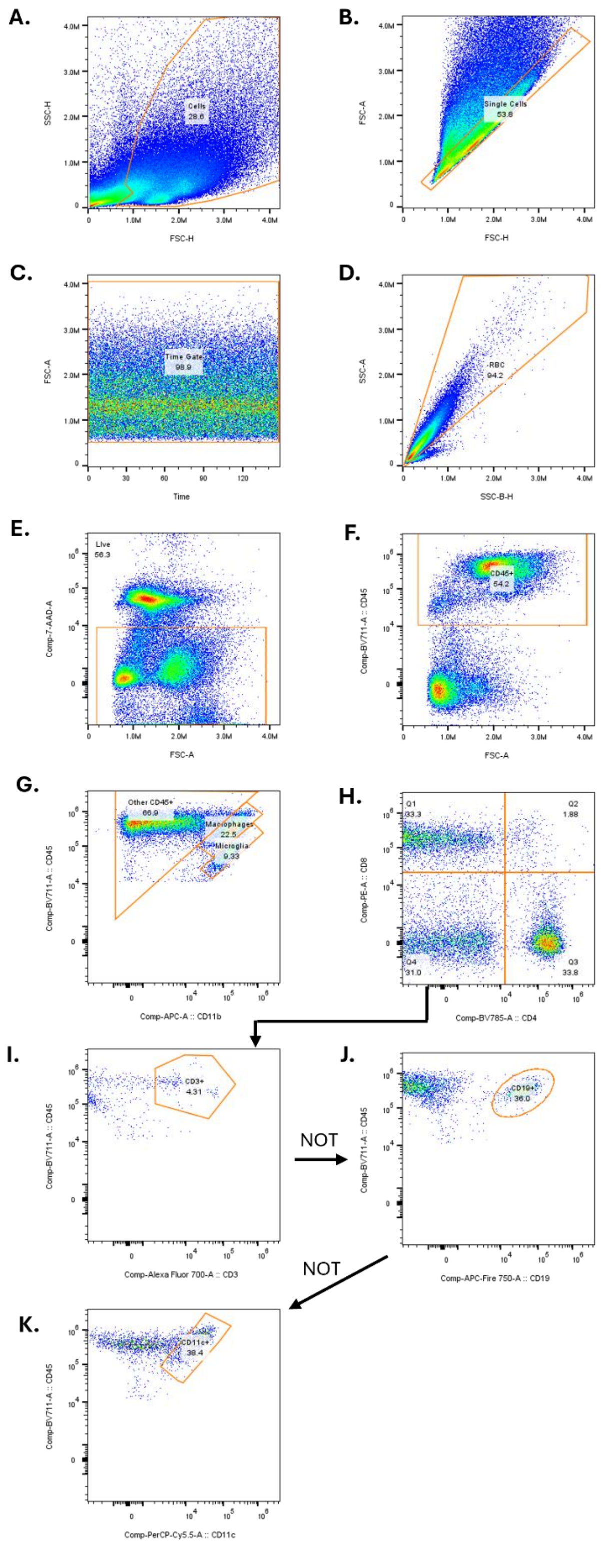

Figure S4. Gating Strategy for AAV treated Rpgr and WT samples. *Gating was performed subjectively on multicolor stained retinal samples from Rpgr and WT animals. The same gates were applied across all samples. In plots A-H the gated population was brought forward into the nest consecutive gate. In plots I gating was performed on the population in the CD4-CD8- 'Q4' gate from plot H as indicated. Plot J was taken from a 'NOT' gate of plot I. Plot K was taken from a NOT gate of plot J. Gate statistics shown represent the proportion of plotted events within each gate. Gating analysis was performed in FlowJo v10.10.0*

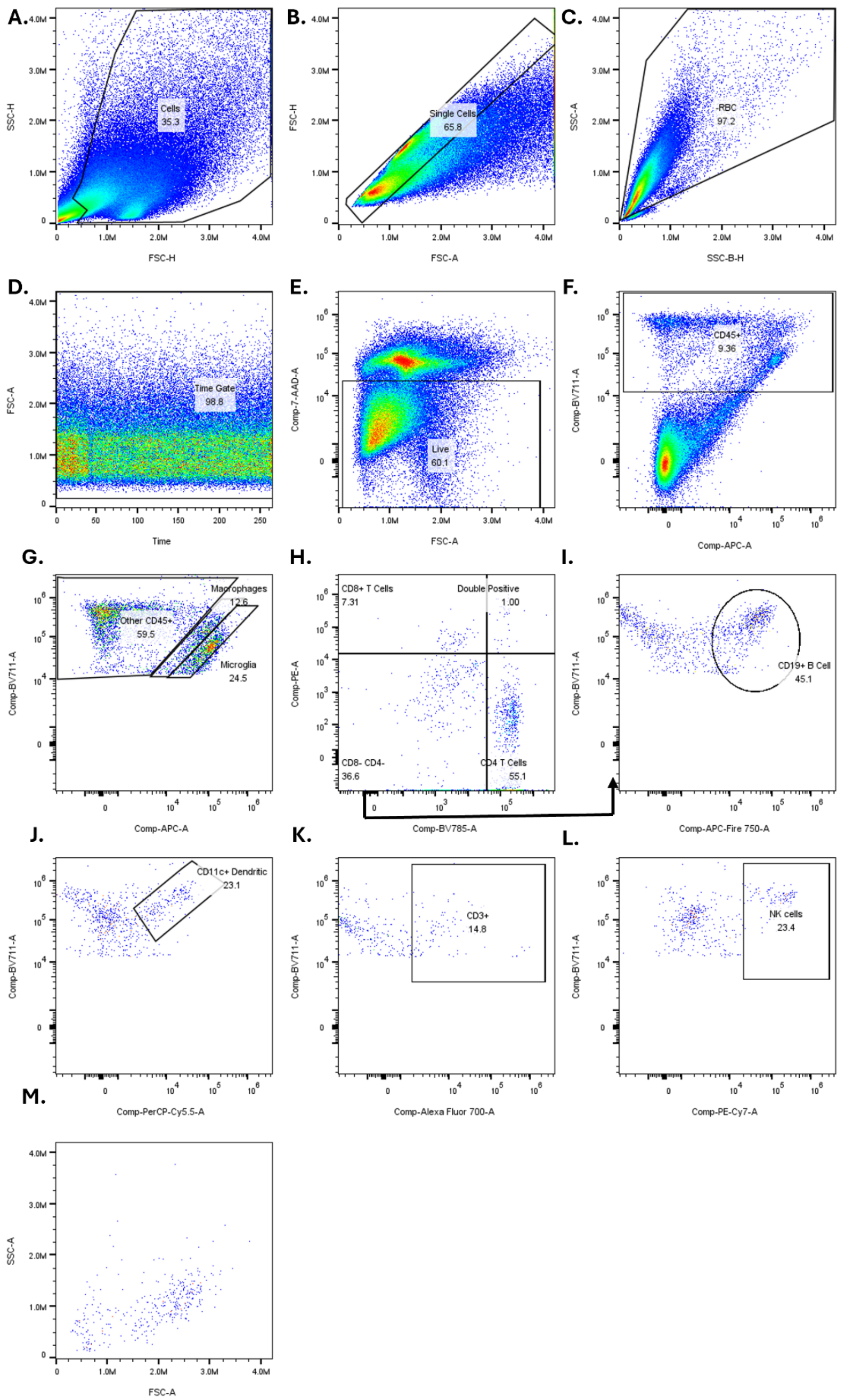

Figure S5. AAV Treated P23H Immunotyping Gating Strategy. *Gating was performed subjectively on multicolor stained retinal samples from AAV or PBS treated retinas from P23H and WT animals. The same gates were applied across all samples. In plots A-H the gated population was brought forward into the next consecutive gate. In plot I gating was performed on the population in the CD4-CD8- 'Q4' gate from plot H as indicated. Plots J-M were taken from a 'NOT' gate of the gated populations on preceding plot. Plot M gives the forward and side scatter profile of untyped CD45 positive cells that did not fit other gates. Gate statistics shown represent the proportion of plotted events within each gate. Gating analysis was performed in FlowJo v10.10.0*

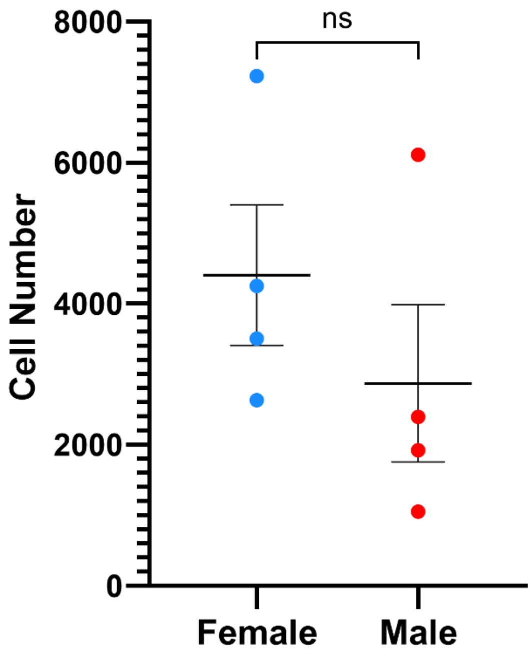

Figure S6. Sex effects in CD45⁺ cells in AAV2.CAG.RHO.GFP treated wild-type mice. *CD45^{+} cells detected in AAV treated retina from the wild-type cohort in the Rho^{P23H} AAV response experiment split by subject sex. Similar analysis was not possible in the Rho^{P23H} cohort due to a more skewed ratio between sexes.*

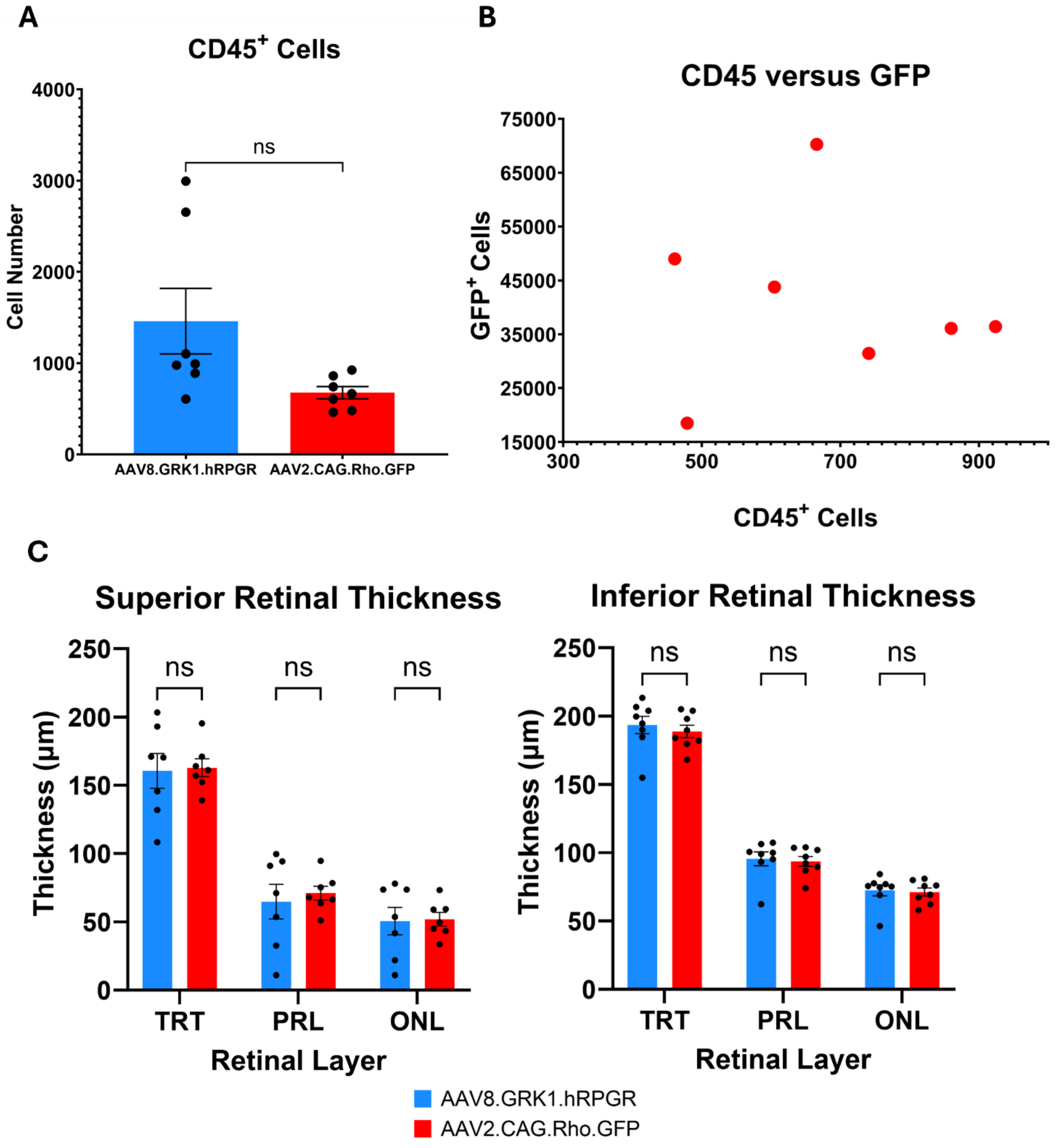

Figure S7. Paired eye comparison of vectors. *9-week-old wild type mice (n=7) received paired eye injections of 1.5x109gc of each vector, with immune infiltration assessed by SLO/OCT imaging at 3 weeks post injection, and eyes harvested for multicolour flow cytometry analysis at 3 weeks post injection. No significant difference was seen in total CD45+ cell populations in treated retinas between the two vectors (p= 0.0625, Wilcoxon matched-pairs signed rank test). CD45+ cells additionally did not correlate with GFP+ cells in AAV2.CAG.RHO.GFP treated retina. There was no significant difference seen in retinal thickness across layers between different vector-treated eyes (Total retinal thickness (TRT), Photoreceptor layer (PRL), Outer nuclear layer (ONL)).*
